# Comprehensive Characterization of Global Barley (*Hordeum vulgare* L.) Collection Using Agronomic Traits, β-Glucan Level, Phenolic Content, and Antioxidant Activities

**DOI:** 10.3390/plants13020169

**Published:** 2024-01-08

**Authors:** Kebede Taye Desta, Yu-Mi Choi, Hyemyeong Yoon, Sukyeung Lee, Jungyoon Yi, Young-ah Jeon, Xiaohan Wang, Jin-Cheon Park, Kyeong-Min Kim, Myoung-Jae Shin

**Affiliations:** 1 National Agrobiodiversity Center, National Institute of Agricultural Sciences, Rural Development Administration, Jeonju 54874, Republic of Korea; 2 International Technology Cooperation Center, Technology Cooperation Bureau, Rural Development Administration, Jeonju 54875, Republic of Korea; 3 National Institute of Crop Science, Rural Development Administration, Wanju 55365, Republic of Korea

**Keywords:** agronomical traits, antioxidant activity, β-glucan, diversity, *Hordeum vulgare*, phenolic content

## Abstract

This study characterized the diversity of 367 barley collections from 27 different countries, including 5 control cultivars, using several phenotypic traits. Morphological traits, including spike type, grain morphology, cold damage, and lodging rate, exhibited wide variations. Eighteen accessions matured early, while four accessions had longer culm and spike lengths than the controls. The ranges of total phenolic content (TPC), β-glucan content, ABTS^•+^ scavenging activity, DPPH^•^ scavenging activity, and reducing power (RP) were 1.79–6.79 mg GAE/g, 0.14–8.41 g/100 g, 3.07–13.54 mg AAE/100 g, 1.56–6.24 mg AAE/g, and 1.31–7.86 mg AAE/g, respectively. Betaone, one of the controls, had the highest β-glucan content. Two accessions had β-glucan levels close to Betaone. Furthermore, 20 accessions exhibited increased TPC compared to the controls, while 5 accessions displayed elevated ABTS^•+^ scavenging activity. Among these, one accession also exhibited higher DPPH^•^ scavenging activity and RP simultaneously. Based on the statistical analysis of variance, all the quantitative traits were significantly affected by the difference in origin (*p* < 0.05). On the other hand, grain morphology significantly affected biochemical traits. Multivariate analysis classified barley accessions into eight groups, demonstrating variations in quantitative traits. There were noteworthy correlations between biochemical and agronomical traits. Overall, this study characterized several barley varieties of different origins, anticipating future genomic research. The barley accessions with superior performances could be valuable alternatives in breeding.

## 1. Introduction

Barley (*Hordeum vulgare* L.) is one of the highly valued crops that have been used as a primary food source for humans as well as livestock. The fact that barley is extensively used in both human and animal diets emphasizes its significance in the global food supply chain [1,2,3]. Additionally, it plays a critical role in the brewing and distilling industries, making a significant contribution to the global alcoholic beverage market [1]. The crop is primarily grown in temperate areas, with the current major producing countries being Russia, Australia, France, and Germany [4].

Barley grains contain a variety of nutrients, including dietary fiber, β-glucan, vitamins, and minerals, among others [5]. Barley also possesses functional phytochemicals, including flavonoids, lignans, phytosterols, and phenolic acids [6,7]. Due to its rich content of nutritional compounds and functional metabolites, barley plays a crucial role in promoting numerous health benefits [8]. For example, the dietary fiber in barley is known for its ability to lower cholesterol, promote bowel movements, and prevent constipation, thereby improving digestive health. Additionally, barley β-glucans can help reduce levels of low-density lipoprotein cholesterol (bad cholesterol) in the bloodstream and decrease the risk of heart disease [6,7,8]. The functional metabolites also possess anti-inflammatory and antioxidant properties, which protect cells against oxidative stress and potentially reduce the likelihood of developing chronic conditions such as cancer [9]. Furthermore, the low glycemic index of barley makes it a suitable choice for individuals with diabetes as it helps regulate blood sugar levels [7]. Generally, the synergistic action of barley metabolites improves overall health and contributes to the crop’s reputation as a nutritious and health-promoting crop [7,8].

As the global population continues to grow, the role of barley in ensuring food security and addressing climate change is of utmost importance [10]. Efforts in breeding have made significant advancements in enhancing barley traits to meet the changing demands of consumers and environmental challenges. Scientists are constantly utilizing genomic sequencing and molecular breeding techniques to unlock the genetic potential of barley. Such studies lead to the development of more productive, disease-resistant, stress-tolerant, and nutritious varieties [11,12]. To support these research advancements, it is crucial to continuously explore the genetic variation found within barley genetic resources. In particular, analyzing the agro-morphological characteristics and biochemical traits of barley genetic resources is essential for the success of barley breeding programs [11,12,13,14]. The agro-morphological features provide valuable information about how different types of barley adapt to different climate conditions and their potential for increasing yield [13,15]. By identifying and recording these features, breeders can develop more productive and sustainable barley varieties with desirable properties [16]. Additionally, understanding the biochemical composition of barley gives insights into the nutritional properties and potential uses of superior genotypes for breeding programs [12]. In general, the continuous assessment of barley genetic resources using agro-morphological and biochemical traits improves the accuracy and effectiveness of breeding endeavors, ultimately leading to the selection and development of high-quality barley varieties [12,14]. Gene banks have a significant role to play in this aspect as they preserve and record a vast number of genotypes with a wide range of genetic variants [17,18].

Previously, several studies have reported the genetic diversity found in barley resources by examining their agricultural, morphological, and biochemical traits [19,20,21,22,23,24]. Nevertheless, there appears to be a lack of research focusing on the diversity of barley resources from different origins. Moreover, only a limited number of studies have combined the assessment of both agro-morphological and biochemical traits to comprehensively analyze the genetic diversity within barley genotypes. Our gene bank at the National Agrobiodiversity Center (Jeonju, Republic of Korea) holds diverse barley genotypes for distribution, conservation, and breeding. However, the characteristics and performances of these genotypes have not been fully studied. Accordingly, this study aimed to evaluate the diversity of 367 barley genetic resources collected from 27 different countries by analyzing their agro-morphological and biochemical characteristics. Additionally, the performance of these resources was compared to five commonly cultivated Korean barley cultivars (Betaone, Saechalssalbori, Heukbori, Heukdahyang, and Saessalbori). The effect of origin on agronomical and biochemical traits was also statistically analyzed in the study, along with the influence of grain morphology on biochemical traits. The results of this research could provide valuable knowledge and evidence regarding the diversity of barley genotypes, shedding light on the impact of their origin. Such understandings might contribute to the selection of superior genotypes and the opportunity to conduct molecular investigations in the future.

## 2. Materials and Methods

### 2.1. Grain Collection, Cultivation, and Preparation

**Figure 1:**
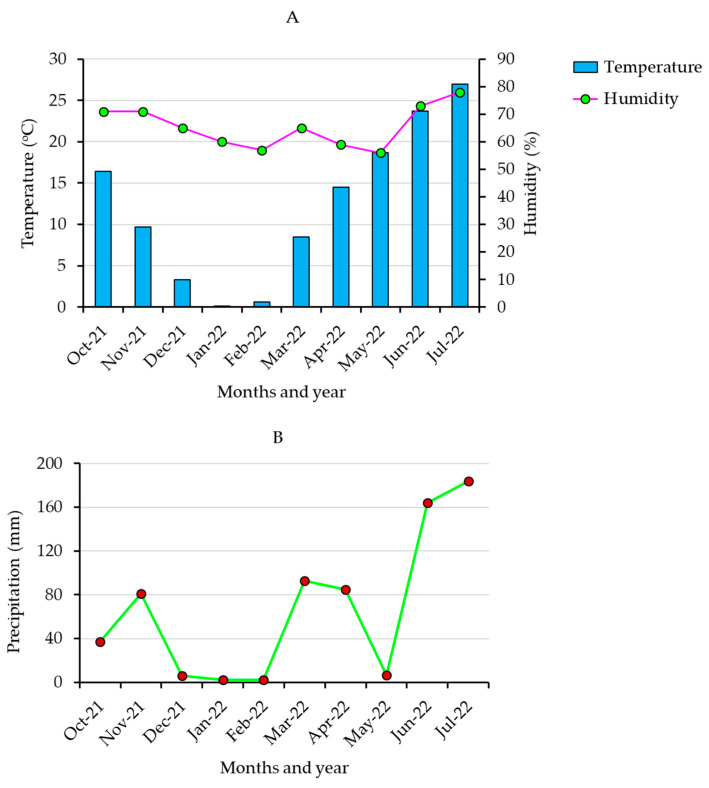
Monthly average temperature and humidity (**A**) and precipitation (**B**) during the growing period in the cultivation area.

In this study, 367 barley accessions originally collected from 27 different countries (Table S1) were obtained from the National Agrobiodiversity Center, Rural Development Administration (RDA Jeonju, Republic of Korea). Five popular Korean barley cultivars (Betaone, Saechalssalbori, Heukbori, Heukdahyang, and Saessalbori) were also used as controls for this study. Field cultivation was conducted during the 2021–2022 winter season on clay loam soil in an experimental farm found at the National Agrobiodiversity Center (RDA Jeonju, Republic of Korea). Briefly, for each genotype, fifty seeds were sown on 26 October 2021, in a 90 cm long row with an accession-to-accession distance of 60 cm. The distance between rows was also kept at 60 cm. A completely decomposed manure was applied as fertilizer (1.5 ton/10 a). The accessions were cultivated under uniform growth conditions, and the cultivation period lasted until July 2022. The average temperature, humidity, and precipitation during the cultivation period are shown in Figure 1. Matured grains were hand-harvested, and samples from each accession were ground by an automated grinder (2010 GEno Grinder), sieved (0.5 mm), and stored at 4 °C pending further analysis.

### 2.2. Recording of Agronomical Traits

The RDA guidebook, which contains details about the characterization and management of genetic resources, was used to document the agronomic qualities of the barley accessions [25]. A total of 15 agronomical traits, consisting of 7 qualitative traits (growth angle, flag leaf angle, spike erectness angle, cold damage, lodging rate, spike type, and grain type) and 8 quantitative traits (days to heading (DH), days to maturity (DM), days from heading to maturity (DHM), culm length (CL), spike length (SL), awn length (AL), number of grains per panicle (GPP), and one-thousand-grain weight (TGW)) were recorded from field observation and post-harvest investigation. In summary, the statuses of the growth angle and flag leaf angle were measured on a 1–3 scale, while the status of the spike erectness angle was measured on a 1–5 scale (Table 1). Likewise, the status of cold damage and lodging rate were each measured on a 1–9 scale. On the other hand, spike type and grain type were visually inspected and categorized. DH was determined by counting the number of days from the moment of sowing until 40% of the plants showed the first spikes. DM was determined as the number of days from sowing to when all the nodes turned yellow. AL represents the length of the longest awn found in a spike. CL was measured by determining the distance from the ground to the bottom of the spike when it reached maturity, while SL was measured by excluding the awns. Likewise, GPP represents the count of grains found in a panicle. TGW was measured by weighing 1000 grains after they were manually cleaned and dried. AL, CL, SL, and TGW values were determined from triplicate measurements, while the GPP value was determined from quintuplet measurements.

### 2.3. Determination of β-Glucan Content

The β-glucan content was determined using an assay kit (Megazyme International Ltd., Bray, Ireland) according to the manufacturer’s guideline, which follows the AOAC method (Method 995.16) [26]. Briefly, 100 mg of ground barley flour was placed in a 15 mL extraction tube and mixed with 200 μL of 50% (*v*/*v*) aqueous ethanol. Subsequently, 4 mL of sodium phosphate buffer solution (20 mM, pH 6.5) was added, and the mixture was incubated for 1 h with the addition of lichenase enzyme (200 μL, 50 U/mL). Following that, the enzymatic reaction was stopped by adding 5 mL of sodium acetate buffer solution (200 mM, pH: 4.0). After centrifugation, 100 μL of the sample was completely reacted with 100 μL of the β-glucosidase enzyme. The concentration of glucose was measured by assessing absorbance at 510 nm using 3 mL of glucose oxidase/peroxidase-developing reagent. The β-glucan content of each variety of barley was then determined as g/100 g from triplicate measurements.

### 2.4. Extraction and Determination of Total Phenolic Content and Antioxidant Activities

#### 2.4.1. Sample Extraction

Sample extraction for the determination of the total phenolic content (TPC) and antioxidant activities was conducted according to our recently reported protocol with some modifications [27]. Specifically, 100 mg of powdered barley grains, in triplicate for each accession, was mixed with 5 mL of 80% aqueous ethanol in 15 mL extraction tube. The mixture was vortexed for about 2 min and sonicated in the dark for 25 min in a water bath set at 25 °C. Then, the mixture was taken off, centrifuged (4000 rpm, 10 min), and the extract was retained. The extraction process was repeated one more time for the residue with the same condition. The combined extract was then stored at 4 °C and used for the analysis of TPC and antioxidant activities within 72 h after extraction.

#### 2.4.2. Determination of TPC and Antioxidant Activities

TPC and antioxidant activities were evaluated using a modified version of our previously published method [27]. In summary, TPC was determined using the Folin–Ciocalteu method and was expressed as milligrams of gallic acid equivalents per gram of dried seed weight (mg GAE/g) using gallic acid as a standard. The antioxidant activities, including DPPH^•^ scavenging activity, reducing power (RP), and ABTS^•+^ scavenging activity, were determined using in vitro colorimetric assays. The DPPH^•^ scavenging activity and RP were determined as milligrams of ascorbic acid equivalents per gram of dried seed weight (mg AAE/g) using ascorbic acid as a standard reference. Additionally, the ABTS^•+^ scavenging activity was calculated as mg AAE/100 g.

### 2.5. Statistical Analysis

All measurements and analysis were conducted in triplicate unless specified, and the results are presented as the average value plus or minus the standard deviation (SD) (mean ± SD). Analysis of variance (ANOVA) was conducted at *p* < 0.05 based on Duncan’s multiple range test to determine significant differences between the measurements followed. All the statistical analyses and principal component analysis (PCA) were computed using xlstat software version 2019.2.2 (Lumivero, CO, USA). Additionally, box plots, scatter plots, and Pearson’s correlation analyses were conducted using R-software (version 4.0.2, r-project). The two-way hierarchical cluster analysis (HCA) was carried out using JMP software version 17 (SAS, Inc., Cary, NC, USA).

## 3. Results and Discussion

### 3.1. Variations of Agro-Morphological Traits

Agro-morphological traits play a crucial role in providing valuable insights into the genetic diversity among different plant genetic materials [13,28]. In this study, a total of 15 agronomical traits were examined, as described before. The barley accessions showed wide variations in both qualitative and quantitative agronomical traits. The qualitative agronomical properties of individual barley accessions can be found in Table S2. Likewise, the numerical values of all quantitative agronomical traits can be read in Table S3. The distributions of the qualitative traits are summarized in Table 1, whereas the statistics of the quantitative traits are presented in Table 2.

#### 3.1.1. Qualitative Agronomical Traits

The majority of barley accessions (63%) had an intermediate growth angle, followed by erect and prostate growth angles (Table 1). Only Betaone and Saessalbori among the control cultivars had an intermediate growth angle, while the remaining three cultivars had an erect growth angle. The flag leaf angle displayed similar variations, with the barley accessions having semi-erect (42%), erect (39%), or horizontal (19%) angles. Except for Saechalssalbori, all control cultivars had a semi-erect flag leaf angle. The spike erectness angle also exhibited a wide range of variation. Around 29% of barley accessions had a semi-erect spike erectness angle, while 19% had an erect angle. Betaone and Saechalssalbori had an erect spike erectness angle, whereas Heukbori, Heukdahyang, and Saessalbori had a semi-erect angle. Similar distributions were observed for other spike erectness angles, such as horizontal, semi-drooping, and drooping. Growth angle, flag leaf angle, and spike angle are important traits associated with various desirable characteristics of barley genotypes. For instance, an erect leaf angle has long been proposed as a means to increase yield, while a spike angle could reduce fusarium head height, a major disease affecting barley production [29,30,31]. On the other hand, Wendt et al. [32] revealed the impact of culm elongation on grain size and yield. Overall, the different morphological traits observed among the barley accessions could offer various options for selecting genotypes with desired properties. The observed wide variance could also be a valuable input for future molecular-level analysis [20,22,28]. In addition to these morphological traits, the variations in cold damage and lodging rate among the barley accessions were also similarly analyzed. Approximately 43% of the population exhibited semi-resistance (21–40%) to cold damage, while another 37% demonstrated full resistance (<20%). Among the control cultivars, only Saechalssalbori displayed semi-resistance, while the others were resistant (Table 1). Interestingly, three barley accessions, namely B-205 from Portugal, D-55 from Iran, and Chungnamnonsan from Korea, showed no signs of cold damage (0%). On the other hand, three accessions, including Shirok Kapo from India, PI 328521 from Greece, and E 517/3 from Ethiopia, suffered severe damage (>81%) (Table S2). As for the lodging rate, the majority of barley accessions (81%) and all control cultivars experienced no lodging (0%). Although no accessions exhibited severe lodging rates (>81%), two accessions, WIR92 from Mongolia and CI 6221 from Turkey, were susceptible to lodging (61–80%). Obtaining high yields in barley requires resistance to cold damage and lodging, and hence, those accessions with high resistance to cold damage and minimal lodging rates could be valuable resources [33,34].

Barley genotypes can be classified into two categories based on their spike morphology: two-row and six-row types. Studies have shown that such spike morphological variation is attributed to the dominance of a specific gene called *vrs1* in the two-row spike type [35]. Despite their distinct differences, both spike types possess unique characteristics and have been extensively utilized in breeding programs [36,37,38]. In this study, approximately 37% of the barley accessions were found to be two-row, while 63% were six-row, like all the control cultivars. Another classification of barley is based on its grain type, which can either be hulless (naked) barley without a husk or hulled (covered) barley with a husk [39]. It was noted that 67% of the barley accessions belonged to the hulled type, while the remaining 33% were hulless. Among the control cultivars, only Heukdahyang was identified as hulless, with the rest being hulled. Once again, the diversity observed in spike morphology and grain type among the barley accessions could help in selecting desirable genotypes for future investigations. Researchers have suggested that the existence or non-existence of a husk in barley might have a significant influence on the concentrations of metabolites across various genotypes [40,41].

#### 3.1.2. Quantitative Agronomical Traits

Variations were also observed in quantitative traits among the barley accessions (Table 2 and Table S3). The coefficient of variation (CV) was the highest for AL (40.31%), followed by GPP (38.72%), and the lowest for DM (3.07%). In the overall population, DH, DM, SL, and GPP exhibited a positive skewness, whereas the other traits displayed a negative skewness. The kurtosis ranged from the most negative value for GPP to the highest positive value for DM. These findings indicate that not all of the quantitative traits followed a normal distribution. DH and DM were in the ranges of 166–213 and 198–238 days, respectively. Likewise, DHM was in the range of 16–45 days. The observed values were comparable to those from several previous studies. For instance, Mansour et al. [22] reported DH of 99–165 days and DM of 140–185 days across 112 elite barley populations, while Dziurdiak et al. [21] reported much lower DH (52.0–93.0 days) and DM (81.7–117.0 days) ranges across 116 barley accessions of different origins. In another study, DH and DM in the ranges of 65.0–125 and 106.5–114.7 days, respectively, were reported across 316 barley genotypes sown at different times [3]. These observations highlight the wide-ranging DH and DM values, which could be attributed to the differences in growth conditions, years of cultivation, and genotypes, among others [10,28,42,43]. In our study, three accessions, including ST-96 from China, PI 559514 from Nepal, and OUI 426 from India, equally displayed the shortest DH, while accession 302 from Turkey took the longest DH (Table S3). ST-96 was also the earliest to mature, while PI 304357 from Portugal and Castelar-034 from Argentina, along with accession 302, were the latest to mature. Among the control cultivars, Betaone was the latest to mature, taking 215 days, while Heukdahyang was the earliest, taking 206 days (Table S3). Early-maturing barley varieties are beneficial for use in breeding as well as dissemination to farmers [44]. Overall, a total of 18 accessions were identified as early maturing compared to all the control cultivars (Table S4). Therefore, these early maturing accessions could be ideal resources in this regard.

The other quantitative agronomical traits also showed variations among the barley accessions. CL and SL were in the ranges of 23.23–127.33 and 3.00–14.40 cm, respectively, while AL ranged from 0.00 to 21.30 mm, the latter showing the highest coefficient of variation (CV: 40.31%) among all quantitative traits. GPP and TGW are other yield-related traits, and each showed wide variations among the barley accessions. The former ranged from 10.40 to 67.40 and the latter from 3.00 to 68.00 g. Among all the barley accessions, B-205 from Portugal and Gyeongnamyang-1985-917 from Korea displayed the shortest CL and SL, respectively, while RNB-9 from Nepal and GRA2256 from Tajikistan had the largest CL and SL, respectively. Interestingly, seven accessions, including PI 510558, PI 270747, and PI 270611 from Peru, WIR1089 from Mongolia, 2155A from Switzerland, Shargudik-2 from India, and RNB-132 from Nepal, displayed no awns with an AL of 0.00 cm. In contrast, CI 6222 from Turkey and HVS 355 from Lebanon equality displayed the longest AL. Previously, several studies have evaluated the variations of quantitative agronomical traits and reported wide-ranging values. A comparable SL range (4.32–11.55 cm), but a much lower AL range (0.92–5.75 cm) were reported across 417 barley accessions grown in Germany [45]. In another study, CL ranged from 53.9 to 82.4 cm, and SL ranged from 49.6 to 90.7 mm [46]. Verma et al. [3] also reported SL ranging from 4.5 to 13.1 cm, which was comparable to our results. Regarding GPP and TKW, Pasam et al. [20] reported a TKW ranging from 17.77 to 67.23 g across 224 barley accessions of worldwide origin, while Dziurdiak et al. [21] reported a GPP ranging from 7.7 to 43.6, both being close to the ranges observed in our study. TKW ranging from 30.5 to 58.8 g, and from 23.2 to 52.0 g were also reported, each being a much lower range compared to our findings [21,22]. In general, these reports along with our results indicate the wide-ranging values of yield and growth-related traits in barley. Once again, differences in environmental, genetic, and post-harvest handling processes could cause such variations [10,28,42,43].

The performance of the barley accessions was also compared to that of the control cultivars. Among the control cultivars, Betaone simultaneously displayed the longest CL and AL, while Heuknuri had the longest SL. In contrast, Saessalchalbori simultaneously displayed the shortest CL and SL and the lowest GPP and TGW, while Saessalbori had the shortest AL and the highest GPP. Heukdahyang displayed the highest GPP. Compared to all the control cultivars, the majority of the accessions (*n* = 223) had higher CL (>72 cm). In particular, 11 accessions were found to have a much longer CL (>100 cm). Likewise, 225 accessions displayed higher SL than all the control cultivars (>5.9 cm), out of which 25 had much higher SL (>10 cm). Interestingly, four accessions, including GRA2256 from Tajikistan, GRA1015 from Ukraine, GRA2621 from Greece, and PI 573706 from Georgia, simultaneously displayed long CL and SL (Table S2). On the other hand, 86 accessions had longer AL than all the control cultivars (>13.7 mm), out of which four (HVS 355, HV S366, and PI 466252 from Lebanon, and CI 6222 from Turkey) had much longer awns (>20 mm). Similarly, 43 accessions displayed a higher GPP than all control cultivars, out of which 10 had a much larger GPP (>60). In terms of TGW, a large population of barley accessions (*n* = 178) outperformed all the cultivars. Out of these, 12 accessions were found to have a much higher TGW (≥62.00 g). Apart from their relation to yield, GPP, TGW, and culm and spike-related characters are important traits that determine the various favorable characteristics of barley genotypes. For instance, shorter culms are found to have increased support and reduced lodging [47]. Likewise, awns are also found to improve seed size and TGW, although they reduce GPP and total yield [48,49]. Therefore, those accessions with favorable properties and superior performances to the cultivars could be utilized in different ways to reduce lodging, maximize grain size, and improve yield (Table S4). Moreover, the identification of genes that are involved in the regulation of grain size, grain weight, and grain number is a research focus, and hence, the observation of wide-ranging values in this study could be a great input for future genomic studies [32].

### 3.2. Variations of Biochemical Traits

#### 3.2.1. β-Glucan and Total Phenolic Contents

Studies showed that β-glucan and phenolic compounds in barley have synergistic roles as antioxidants to prevent various ailments. Therefore, they are one of the determinant factors in the selection of quality barley genotypes [50]. In this study, the β-glucan content in the entire population ranged from 0.14 to 8.41 g/100 g, showing a sixty-fold variation (Table 2 and Table S3). Betaone, one of the control cultivars, showed the maximum β-glucan content, while Castelar-668 from Argentina had the lowest (*p* < 0.05). Previously, β-glucan content in the ranges of 2.40–7.42 g/100 g was reported across barley genotypes grown in the Czech Republic and Spain [23], while Hang et al. [24] reported 4.20–8.94% across barley genotypes grown at three different locations in the USA, both being close to the ranges observed in our study. In another study, a much narrower range of β-glucan content (2.5–4.6%) was reported in barley-inbred lines grown in Italy [51]. Like in the agronomical traits, both environmental and genetic factors contribute to the variations observed in biochemical traits in barley, resulting in such wide-ranging values [52]. A comparative analysis of the barley accessions to the control cultivars revealed that none of the barley accessions had a higher β-glucan content than Betaone. Overall, the average β-glucan content was significantly higher in the control cultivars. Although the majority of the barley accessions (*n* = 203) displayed a lower β-glucan content than all the control cultivars, 79 accessions had a higher β-glucan content than all the control cultivars except Betaone. In particular, accessions PI235639 from Germany and IG38956 from Israel displayed β-glucan levels (7.67 and 7.08%, respectively) close to that of Betaone and, hence, could be good sources of β-glucan [53].

The TPC was also in the range of 1.79–6.97 mg GAE/g, showing an approximately four-fold variation (Table 2 and Table S3). The highest TPC was found in accession K222037-2 from Turkey, while the lowest TPC was found in accession GAW 72-11 from Ethiopia. Several studies have examined the TPC levels of different barley genotypes along with their antioxidant activities. However, differences in cultivation conditions, use of biostimulants, and experimental parameters influence the levels of these biochemical traits, resulting in wide differences in the reported values [39,40,54]. For instance, a TPC ranging from 333.9 to 460.8 mg GAE/100 g was reported across four Chinese barley varieties [39]. Likewise, TPC ranged from 1929.0 to 2917.0 µg GAE/g in Iannucci et al.’s [51] study, which was comparable to the result obtained in our study. In contrast, a much narrower TPC range (195.02–220.11 mg GAE/100 g) compared to our findings was reported across four barley cultivars grown in Tunisia [55]. Among the control cultivars, Betaone once again displayed the highest TPC (4.00 mg GAE/g), while Saessalbori displayed the lowest (2.91 mg GAE/g). A total of 20 accessions displayed a higher TPC compared to all the controls, including Betaone, while 194 accessions had a lower TPC. As highlighted before, β-glucan and phenolic compounds play significant roles in disease prevention and health promotion. Moreover, the development of barley varieties with maximized levels of these metabolites is one of the research focuses [12,53,56,57]. Accordingly, those accessions that displayed higher levels of TPC and β-glucan content could be important genetic materials (Table S4).

#### 3.2.2. Antioxidant Activities

Excess production of reactive nitrogen species (RNS) and reactive oxygen species (ROS) in the human body is the cause of many health ailments [7,12,57]. Dietary crops that have the property to scavenge these reactive radicals are very important and are of research focus [12]. In this study, the antioxidant activities of the barley accessions were determined using three in vitro radical scavenging assays, including DPPH^•^ scavenging activity, ABTS^•+^ scavenging activity, and reducing power (RP). Significant variation (*p* < 0.05) was observed among the barley accessions, and their performance also significantly differed from that of the control cultivars (Table 2 and Table S3). DPPH^•^ scavenging activity and RP were in the ranges of 1.56–6.24 and 1.31–7.86 mg AAE/g, respectively, the former showing a four-fold difference while the latter showing a six-fold difference. ABTS^•+^ scavenging activity was in the ranges of 3.07–13.54 mg AAE/100 g, showing an approximately four-fold variation. GRA2621, a barley accession from Greece, simultaneously displayed the highest DPPH^•^ scavenging activity, ABTS^•+^ scavenging activity, and RP. In contrast, accessions PI 477805 from Peru, CIho 4169 from Afghanistan, and Clho 3970-1 from Mongolia displayed the lowest ABTS^•+^ scavenging activity, DPPH^•^ scavenging activity, and RP, respectively. Several studies have evaluated the antioxidant activities of barley genetic materials. For instance, Suriano et al. [58] studied 20 barley genotypes and reported DPPH^•^ scavenging activity and ABTS^•+^ scavenging activity in the ranges of 8.20–13.40 and 10.51–15.60 µmol Trolox equivalent per gram (µmol TE/g), respectively. In another study, DPPH^•^ scavenging activity ranged from 9.33 to 11.78 µmol TE/g, while ABTS^•+^ scavenging activity ranged from 11.39 to 13.58 µmol TE/g [59]. Salar et al. [60] also reported DPPH, ABTS, and RP in the ranges of 45.22–59.84, 43.22–64.35, and 22.55–77.58%, respectively. Apart from genotype and environmental effects, discrepancies in sample extraction protocols, assays, and reporting methods could cause such variations, as highlighted before [6,61]. In the control cultivars, each of the DPPH^•^ scavenging activity and ABTS^•+^ scavenging activity decreased in the order of Betaone > Saechalssalbori > Heukdahyang > Saessalbori > Heuknuri.

In the RP, however, Saessalbori and Heuknuri interchanged their orders, the remaining being similar to those observed for DPPH^•^ scavenging activity and ABTS^•+^ scavenging activity. A comparative analysis revealed that the majority of the barley accessions had much lower antioxidant activities than all the control cultivars. Specifically, a total of 245 accessions had lower ABTS, 299 accessions had lower DPPH, and 319 accessions had lower RP than all the control cultivars. Despite these observations, five accessions, including GRA2621 from Greece, GRA2256 from TJK, UZB-BHJ-2002-23-2 from Uzbekistan, PI 204875 from Turkey, and GRA1015 from Ukraine, displayed higher ABTS^•+^ scavenging activity than all the control cultivars. In contrast, only one accession, GRA2621, from Greece displayed higher DPPH^•^ scavenging activity. Once again, GRA2621 and GRA2256 from TJK were the two accessions that displayed higher RP than all the control cultivars (Table S4). Interestingly, all of these accessions simultaneously displayed a higher TPC level than all the control cultivars, and hence, they could be important sources of antioxidants [6,12,57].

### 3.3. Effect of Origin on Quantitative Traits

**Figure 2:**
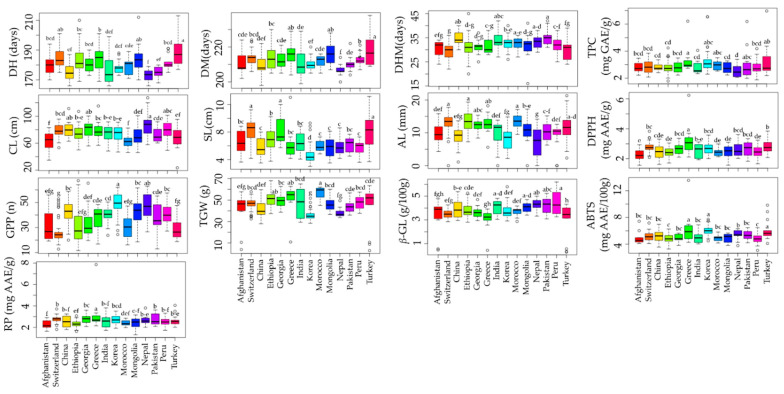
Effect of origin on agronomical traits and biochemical traits in barley accessions grown in Korea. Different letters on boxplots indicate significantly different means based on Duncan’s multiple range test (*p* < 0.05). ABTS: ABTS^•+^ scavenging activity, AL: Awn length, CL: Culm length, DH: Days to heading, DM: days to maturity, DHM: Days from heading to maturity, DPPH: DPPH^•^ scavenging activity, GPP: Number of grains per panicle, RP: Reducing power, SL: Spike length, TGW: One-thousand grains weight, TPC: Total phenolic content, β-GL: β-glucan content.

The effect of origin on agro-morphological and biochemical traits has been investigated in various crops [62]. Although different studies have evaluated the agro-morphological and biochemical traits of global barley collections, the effect of origin has been rarely investigated [20,21,23]. In this study, a total of 14 countries with accession numbers >10 and accounting for 91.83% of the total population studied were used to view the effect of origin on each parameter investigated. Our results found significant variation in all of the agronomical as well as biochemical traits analyzed (Figure 2 and Table S5). Nepal accessions were found to be early maturing with an average DM of 206.57 days while Turkey accessions were late maturing with an average DM of 219 days (*p* < 0.05). Accessions from Nepal and Turkey also displayed the highest average CL (88.25 cm) and SL (8.22 cm), respectively, significantly different from most other countries. In contrast, the lowest average CL (62.02 cm) and SL (4.73 cm) values were in accessions from Morocco and Korea, respectively. Korean accessions also displayed the highest average GPP (49.26) and the lowest average TGW (35.88 g) (*p* < 0.05). In contrast, the lowest average GPP (26.36) was found in accessions from Switzerland, while the highest average TGW (56.35 g) was observed in Moroccan accessions (*p* < 0.05). Regarding the biochemical traits, the average β-glucan content was the highest in accessions from Peru (4.45 g/100 g) and the lowest in accessions from Turkey (2.99 g/100 g) (*p* < 0.05). On the other hand, accessions from Greece simultaneously displayed the highest average TPC (3.32 mg GAE/g), DPPH^•^ scavenging activity (3.22 mg AAE/g), ABTS^•+^ scavenging activity (6.34 mg AAE/100 g), and RP (3.21 mg AAE/g). Accessions from Nepal, on the other hand, displayed the lowest average TPC (2.52 mg GAE/g), while those from Afghanistan displayed the lowest average DPPH^•^ scavenging activity (2.24 mg AAE/g) and RP (2.27 mg AAE/g). The lowest average ABTS^•+^ scavenging activity was found in accessions from Peru (4.78 mg AAE/100 g). Overall, our results showed that origin had a significant effect on agronomical and biochemical traits and, hence, could be a determinant factor in the selection of barley genotypes. The effect of origin could be attributed to the difference in geological conditions, which impacts the chemical composition of plants over time [62]. Additionally, it was discovered that origins with higher TPC exhibited comparatively higher levels of antioxidant activities, which further emphasizes the role of barley polyphenols in regulating reactive radicals [6,7,8].

### 3.4. Effect of Grain Type on Biochemical Traits

According to grain type, barley could be either hulless (naked) barley without a husk, or hulled (covered) barley with a husk [39]. The presence or absence of husk is thought to influence the composition and levels of biochemical traits in barley genotypes [40,41]. As described before, the barley accessions in our study were also either hulless or hulled (Table 1), and the variations in biochemical traits between them were statistically analyzed. The result showed that hulless genotypes had significantly higher average β-glucan content (4.24 g/100 g) than the hulled genotypes (3.42 g/100 g) (*p* < 0.05) (Table 3).

β-glucans are thought to be found in the cell wall of barley endosperm [53]. The absence of husk in the hulless genotypes could have exposed the grain parts for better extraction of β-glucan. Although there are no comparative studies, some studies also showed a higher level of β-glucan content in hulless barley genotypes than in hulled genotypes [40]. In terms of the other biochemical traits, however, hulled accessions outweighed the hulless accessions. Specifically, the average TPC, DPPH^•^ scavenging activity, ABTS^•+^ scavenging activity, and RP were 3.02 mg GAE/g, 2.68 mg AAE/g, 5.57 mg AAE/100 g, and 2.71 mg AAE/g, respectively, in the hulled barley accessions, each being significantly different from the average values observed in the hulless accessions (*p* < 0.05) (Table 3). Previous studies also indicated the importance of husk in barley as a source of health-promoting metabolites, including polyphenols [40,41]. Overall, our research indicated that the content of metabolites differs among various types of barley grains. Consequently, hulled barley varieties have the potential to provide significant amounts of polyphenols and antioxidants, whereas hulless varieties may be rich sources of β-glucan.

### 3.5. HCA, PCA, and Correlation Analysis

**Figure 3:**
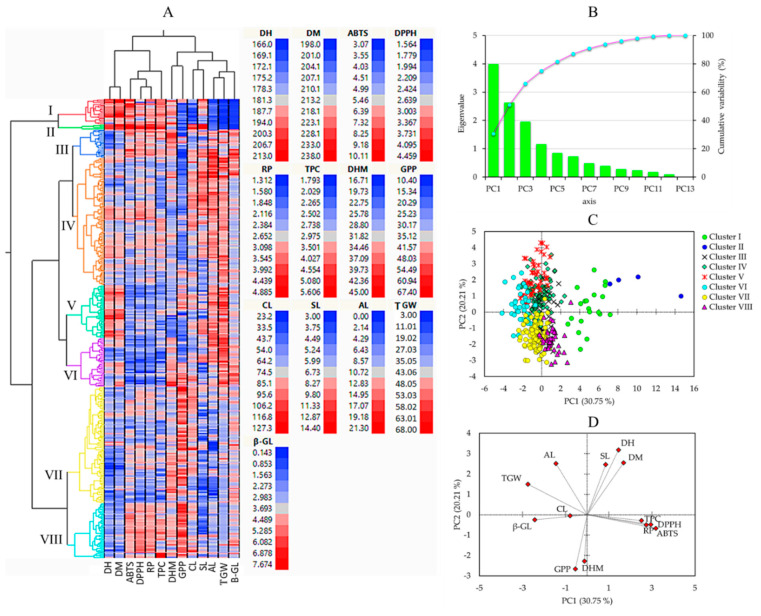
Hierarchical cluster analysis (**A**), scree plot of principal components (**B**), score plot of barley accessions (**C**), and loading plots of variables (**D**) in the PCA. ABTS: ABTS^•+^ scavenging activity, AL: Awn length, CL: Culm length, DH: Days to heading, DM: Days to maturity, DHM: Days from heading to maturity, DPPH: DPPH^•^ scavenging activity, GPP: Number of grains per panicle, RP: Reducing power, SL: Spike length, TGW: One-thousand grains weight, TPC: Total phenolic content, β-GL: β-glucan content.

Multivariate statistical tools, including HCA, PCA, and correlation analysis, aid in comprehending the diversity of plant resources and examining the interaction between phenotypic traits [21,51]. In this study, HCA and PCA were computed using the entire quantitative dataset (Figure 3). The HCA clustered the barley genotypes into eight clusters (Figure 3A).

**Figure 4:**
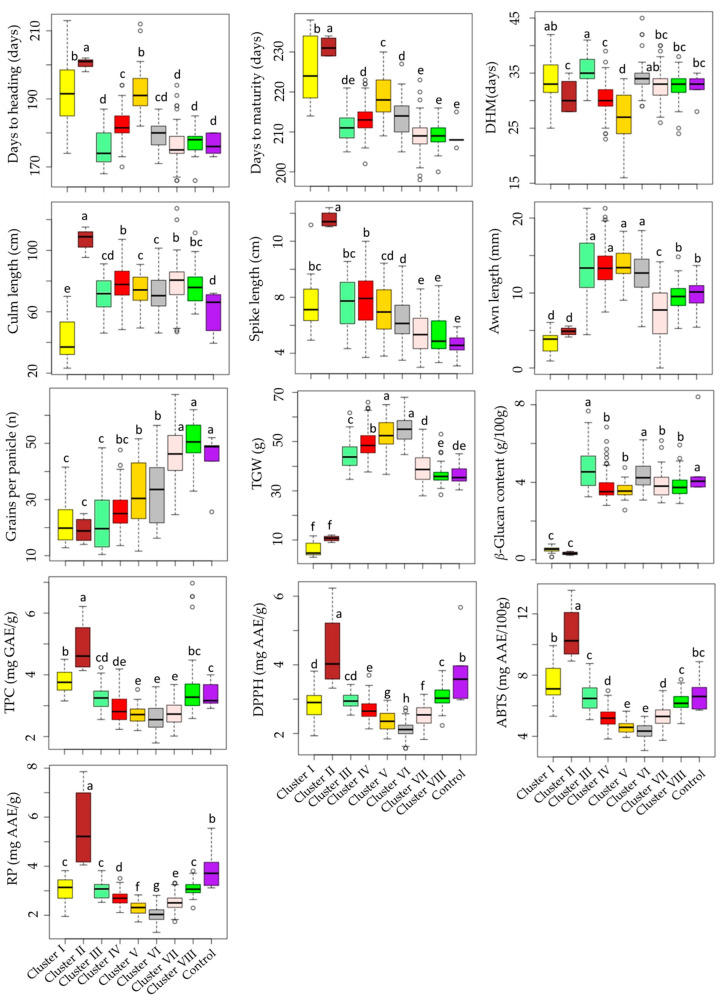
Boxplots showing the variations of quantitative traits across barley accession clusters and compared to the control cultivars. Different letters on boxplots indicate significantly different means based on Duncan’s multiple range test (*p* < 0.05). ABTS: ABTS^•+^ scavenging activity, AL: Awn length, CL: Culm length, DH: Days to heading, DM: Days to maturity, DHM: Days from heading to maturity, DPPH: DPPH^•^ scavenging activity, GPP: Number of grains per panicle, RP: Reducing power, SL: Spike length, TGW: One-thousand-grain weight, TPC: Total phenolic content, β-GL: β-glucan content.

Cluster I had 20 accessions, cluster II had only 4 accessions (including GRA1015, GRA2256, GRA2621, and PI204875), cluster III had 23 accessions, cluster IV had 102 accessions, cluster V had 42 accessions, cluster VI had 39 accessions, cluster VII had 93 accessions, and cluster VIII had 44 accessions. As shown in Figure 4, barley accessions in cluster II were characterized by having the largest average DM (231.25 days), and DH (200.50 days) signifying their late maturing characteristics. In contrast, early-maturing accessions dominated clusters VII and VIII, with an average DM of 209.13 and 209.20 days, respectively. Once again, accessions in cluster II displayed the longest average CL (107.08 cm) and SL (13.56 cm) and the lowest average GPP (19.15), the former two being significantly different from the rest of the clusters and the latter being significantly different from all but clusters I, III, and IV (*p* < 0.05). Accessions in clusters VII and VIII were characterized by having the highest average GPP (46.19 and 50.54, respectively), each significantly different from the remaining clusters (*p* < 0.05), but not from the average GPP observed in the control cultivars (32.06). In terms of biochemical traits as well, accessions in cluster II displayed the highest average TPC (4.89 mg GAE/g), DPPH^•^ scavenging activity (4.40 mg AAE/g), ABTS^•+^ scavenging activity (10.74 mg AAE/100 g), and RP (5.58 mg AAE/g), each being significantly different from the other clusters and also the control cultivars (*p* < 0.05). Despite this, cluster II displayed the lowest average β-glucan content (0.33 g/100 g). Cluster III had the highest average β-glucan content (4.76 g/100 g) next to the control cultivars (4.85 g/100 g). These values were significantly different from the average β-glucan contents found in all other clusters except cluster VI (4.41 g/100 g). Cluster VI was characterized by having significantly lower levels of TPC and antioxidant activities than the other clusters (*p* < 0.05). Specifically, the average TPC in cluster VI (2.58 mg GAE/g) was significantly different from all clusters except clusters IV (2.90 mg GAE/g), V (2.70 mg GAE/g), and VII (2.73 mg GAE/g). Likewise, the average ABTS^•+^ scavenging activity (4.33 mg AAE/100 g) was significantly different from all clusters except cluster V (4.61 mg AAE/100 g). On the other hand, the average DPPH^•^ scavenging activity and RP in cluster VI (2.12 and 2.05 mg AAE/g, respectively) were significantly different from the other clusters. These observations indicate that the accessions in cluster VI exhibited low effectiveness against reactive radicals [12].

PCA was also computed to further view the distribution of the barley accessions and their associations with all the quantitative parameters. The PCA yielded four components with eigenvalues greater than one (Figure 3B). The score plot of the barley accessions and loading plot of variables were computed along PC1 and PC2, which together explained about 51% of the total variance observed. As shown in Figure 3C, the PCA separated the barley accessions according to their cluster. In particular, the four accessions in cluster II were distributed on the positive end of both the PC1 and PC2 axes. As shown in Figure 3D and Table 4, the biochemical traits including TPC, DPPH^•^ scavenging activity, ABTS^•+^ scavenging activity, RP, and β-glucan content were the major contributors along PC1 each, except β-glucan content displaying a positive loading factor (FL) greater than 0.5. β-glucan content was the only biochemical trait with a negative FL greater than 0.5 along PC1. Among the agronomical traits, only TGW had a negative FL higher than −0.5 along PC1. In contrast, the other agronomical traits were the major contributors to PC2. DH, DM, and SL, AL had positive FL above 0.5, while DHM GPP displayed negative FL above 0.5. In general, the PCA supported the HCA observation and signified that quantitative agronomical traits along with biochemical traits could be used to distinguish between large populations of barley genotypes.

**Figure 5:**
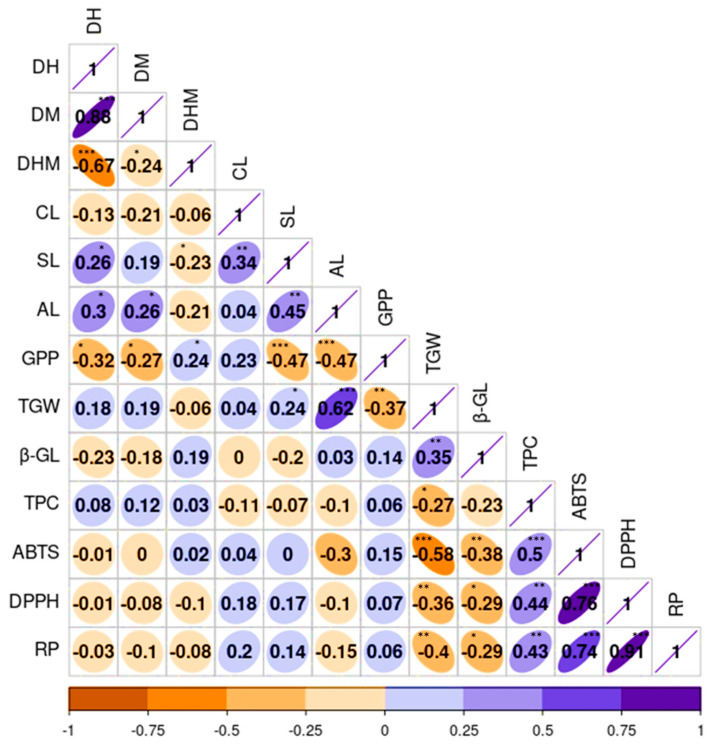
Pearson’s correlation matrix displaying the correlation between quantitative agronomical and biochemical traits in the whole barley population. Axis values represent the range of each trait. ABTS: ABTS^•+^ scavenging activity, AL: Awn length, CL: Culm length, DH: Days to heading, DM: Days to maturity, DHM: Days from heading to maturity, DPPH: DPPH^•^ scavenging activity, GPP: Number of grains per panicle, RP: Reducing power, SL: Spike length, TGW: One-thousand-grain weight, TPC: Total phenolic content, β-GL: β-glucan content. *** *p* < 0.001, ** *p* < 0.01, * *p* < 0.05.

To observe the relationship between quantitative agronomical and biochemical traits, a correlation analysis was also conducted (Figure 5). As depicted in Figure 3D, the results aligned with the findings from the PCA. The growth-related traits, namely DH and DM, displayed a significant and positive correlation with each other (*r* = 0.88, *p* < 0.001). This observation was consistent with the results of numerous previous studies [3,18]. Additionally, SL also exhibited positive correlations with CL (*r* = 0.34) and AL (*r* = 0.45), both being significant at *p* < 0.01. Conversely, GPP demonstrated a negative correlation with SL (*r* = −0.47) and AL (*r* = −0.47) while displaying a positive association with CL (*r* = 0.23), each except the latter being significant (*p* < 0.001). TGW showed positive correlations with AL (*r* = 0.62) and SL (*r* = 0.24) but displayed a negative correlation with GPP (*r* = −0.37) and a weak correlation with CL (*r* = 0.04). Many of these correlations were in agreement with previous studies [3,18,45]. Among the biochemical traits, β-glucan content demonstrated a strong and positive association with TGW (*r* = 0.35, *p* < 0.01), both showing negative and significant correlations with the other biochemical traits. In contrast, TPC displayed positive and significant associations with each of the antioxidant activities, including DPPH, ABTS, and RP (*r* ≥ 0.43). These findings are in line with previous studies, which strengthen the role of phenolic compounds in controlling reactive radicals [55,58,60].

## 4. Conclusions

This study analyzed the diversity within a large collection of barley accessions recently grown in Korea, using both agro-morphological and biochemical characteristics. The results indicated significant differences in agricultural and biochemical traits among these barley accessions. These variations in the agricultural and biochemical aspects of the barley accessions could potentially be used as a basis for further research aimed at identifying the genes responsible for controlling these different traits as well as exploring variations in specific metabolites. Additionally, it was observed that the origin of the barley and the type of grain had a significant impact on the agronomical traits and biochemical parameters, respectively. Therefore, these factors could be used to classify a large population of barley genetic materials based on their phenotypic characteristics. Furthermore, using multivariate statistical techniques, this study identified several superior accessions that performed better than the control cultivars. Therefore, these accessions that have been identified could serve as valuable assets for breeding objectives and dissemination. Future research should prioritize the exploration of additional relevant metabolites, as this particular study only examined the most common biochemical traits. Additionally, there is a significant opportunity to further explore how other characteristics related to grains might influence these biochemical traits.

## Figures and Tables

**Table 1:** Frequency (*f*) and relative frequency (%) of qualitative agronomical traits in global barley accessions cultivated in Korea.

						Controls				
	Value	Scale	Status	*f*	*%*	1	2	3	4	5
Growth angle (°)	61–90	1	Erect	98	27		√	√	√	
	31–60	2	Intermediate	230	63	√				√
	0–30	3	Prostate	39	11					
Flag leaf angle	61–90	1	Erect	143	39		√			
	31–60	2	Semi-erect	155	42	√		√	√	√
	0–30	3	Horizontal	69	19					
Spike erectness angle (°)	61–90	1	Erect	70	19	√	√			
	31–60	2	Semi-erect	108	29			√	√	√
	−30–30	3	Horizontal	62	17					
	−60–(−30)	4	Semi-drooping	62	17					
	−90–(−60)	5	Drooping	65	18					
Cold damage (%)	0	0	Nothing	3	1					
	<20	1	Resistant	135	37	√		√	√	√
	21–40	3	Semi-resistant	156	43		√			
	41–60	5	Intermediate	58	16					
	61–80	7	Susceptible	12	3					
	>81	9	Severe	3	1					
Lodging rate (%)	0	0	Nothing	297	81	√	√	√	√	√
	<20	1	Resistant	33	9					
	21–40	3	Semi-resistant	18	5					
	41–60	5	Intermediate	12	3					
	61–80	7	Susceptible	7	2					
	>81	9	Severe	0	0					
Spike type	-	-	Two rows	134	37					
	-	-	Six rows	233	63	√	√	√	√	√
Grain type	-	-	Hulled (Husked)	246	67				√	
	-	-	Hulless (Non-husked)	121	33	√	√	√		√

Controls: 1: Betaone, 2: Saechalssalbori, 3: Heukbori, 4: Heukdahyang, 5: Saessalbori.

**Table 2:** Statistics of quantitative agronomical characteristics in global barley accessions cultivated in Korea.

Trait	Category	Min	Max	Mean	SD	CV (%)	Skewness	Kurtosis
DH (days)	Population	166.00	213.00	181.35 ^a^	7.94	4.38	1.06	1.54
	Control	173.00	180.00	176.60 ^a^	2.94	1.66		
DM (days)	Population	198.00	238.00	213.18 ^a^	6.55	3.07	1.26	2.24
	Control	206.00	215.00	209.00 ^a^	3.10	1.48		
DHM (days)	Population	16.00	45.00	31.82 ^a^	3.77	11.85	−0.22	1.14
	Control	28.00	35.00	32.40 ^a^	2.42	7.46		
CL (cm)	Population	23.23	127.33	74.50 ^a^	15.01	20.15	−0.50	1.41
	Control	39.50	72.00	59.30 ^b^	13.24	22.33		
SL (cm)	Population	3.00	14.40	6.73 ^a^	2.08	30.93	0.69	0.37
	Control	3.10	5.90	4.58 ^b^	0.93	20.30		
AL (mm)	Population	0.00	21.30	10.72 ^a^	4.32	40.31	−0.37	−0.04
	Control	5.43	13.67	9.78 ^a^	2.71	27.76		
GPP (*n*)	Population	10.40	67.40	35.12 ^a^	13.60	38.72	0.16	−1.05
	Control	25.60	52.00	43.84 ^a^	9.51	21.70		
TGW (g)	Population	3.00	68.00	43.06 ^a^	12.65	29.39	−1.24	2.22
	Control	30.40	45.00	36.76 ^a^	4.96	13.48		
β-GL (g/100 g)	Population	0.14	7.67	3.69 ^b^	1.12	30.44	−0.64	3.48
	Control	3.75	8.41	4.85 ^a^	1.79	36.87		
TPC (mg GAE/g)	Population	1.79	6.97	2.97 ^a^	0.66	22.09	2.32	9.90
	Control	2.91	4.00	3.37 ^a^	0.41	12.10		
ABTS (mg AAE/100 g)	Population	3.07	13.54	5.46 ^b^	1.16	21.23	1.89	7.52
	Control	5.71	8.88	6.84 ^a^	1.16	16.97		
DPPH (mg AAE/g)	Population	1.56	6.24	2.64 ^b^	0.45	17.22	1.84	11.02
	Control	2.98	5.67	3.85 ^a^	0.99	25.62		
RP (mg AAE/g)	Population	1.31	7.86	2.65 ^b^	0.56	21.03	2.87	20.43
	Control	3.12	5.55	3.95 ^a^	0.88	22.33		

Different superscript letters in a column within a category indicate significantly different means based on Duncan’s multiple range test (*p* < 0.05). ABTS: ABTS^•+^ scavenging activity, AL: Awn length, CL: Culm length, DH: Days to heading, DM: days to maturity, DHM: Days from heading to maturity, DPPH: DPPH^•^ scavenging activity, GPP: Number of grains per panicle, RP: Reducing power, SL: Spike length, TGW: One-thousand grains weight, TPC: Total phenolic content, β-GL: β-glucan content.

**Table 3:** Variations of biochemical traits in hulled and hulless barley accessions grown in Korea.

Values	β-GL (g/100 g)		TPC (mg GAE/g)		ABTS (mg AAE/100 g)		DPPH (mg AAE/g)		RP (mg AAE/g)	
	H	NH	H	NH	H	NH	H	NH	H	NH
Min	0.14	0.43	1.79	1.88	3.27	3.07	1.56	1.57	1.63	1.31
Max	7.67	6.19	6.97	6.54	13.54	7.35	6.24	3.83	7.86	3.80
Mean	3.42 ^b^	4.24 ^a^	3.02 ^a^	2.88 ^b^	5.57 ^a^	5.24 ^b^	2.68 ^a^	2.57 ^b^	2.71 ^a^	2.54 ^b^
SD	1.16	0.79	0.64	0.67	1.28	0.82	0.45	0.46	0.58	0.50

Different superscript letters in a row within a category indicate significantly different means based on Duncan’s multiple range test (*p* < 0.05). ABTS: ABTS^•+^ scavenging activity, DPPH: DPPH^•^ scavenging activity, H: Hulled, NH: Hulless, RP: Reducing power, TPC: Total phenolic content, β-GL: β-glucan content.

**Table 4:** Eigenvalues and variability of the first four principal components and contribution of agronomical and biochemical traits in the PCA.

Variables	PC1		PC2		PC3		PC4	
	FL	%	FL	%	FL	%	FL	%
DH	0.39	3.86	0.77	22.67	−0.33	5.46	−0.32	8.65
DM	0.46	5.21	0.62	14.59	−0.44	9.79	−0.01	0.00
DHM	−0.03	0.03	−0.55	11.59	−0.07	0.26	0.66	37.41
CL	−0.21	1.13	−0.01	0.01	0.71	25.60	−0.41	14.67
SL	0.23	1.32	0.60	13.60	0.47	11.17	0.17	2.50
AL	−0.39	3.82	0.61	14.23	0.42	8.89	0.24	5.02
GPP	−0.15	0.54	−0.65	15.84	−0.05	0.13	−0.57	27.97
TGW	−0.74	13.79	0.36	5.06	0.28	4.05	0.03	0.06
ABTS	0.86	18.52	−0.16	1.00	0.21	2.31	0.08	0.62
DPPH	0.74	13.69	−0.12	0.54	0.52	13.95	−0.05	0.19
RP	0.79	15.81	−0.12	0.53	0.48	11.63	−0.04	0.11
TPC	0.68	11.50	−0.07	0.19	0.03	0.06	0.15	2.02
β-GL	−0.66	10.78	−0.06	0.14	0.36	6.71	0.09	0.77
Eigenvalue	4.00		2.63		1.96		1.16	
Variability (%)	30.75		20.21		15.08		8.93	
Cumulative (%)	30.75		50.96		66.04		74.96	

ABTS: ABTS^•+^ scavenging activity, AL: Awn length, CL: Culm length, DH: Days to heading, DM: days to maturity, DHM: Days from heading to maturity, DPPH: DPPH^•^ scavenging activity, FL: Loading factor, GPP: Number of grains per panicle, RP: Reducing power, SL: Spike length, TGW: One-thousand grains weight, TPC: Total phenolic content, β-GL: β-glucan content.

## Data Availability

Data are contained within the article or Supplementary Materials.

## Supplementary Materials

The following supporting information can be downloaded at: https://www.mdpi.com/article/10.3390/plants13020169/s1, Table S1: The frequency of 367 barley accessions according to their origin; Table S2: Qualitative agro-morphological traits in global barley accessions grown in Korea; Table S3: Quantitative agronomical traits and biochemical traits in global barley accessions grown in Korea; Table S4: Average values of top-performing accessions relative to the control cultivars; Table S5: Effect of origin on quantitative agronomical traits and biochemical parameters in global barley accessions grown in Korea.
