# Supplementary material for: Comprehensive Characterization of Global Barley (Hordeum vulgare L.) Collection Using Agronomic Traits, β-Glucan Level, Phenolic Content, and Antioxidant Activities
Source: Plants (Basel). 2024 Jan 8;13(2):169. doi: 10.3390/plants13020169 (PMC10818635; doi:10.3390/plants13020169)
Supplement: Supplementary file 1 [file plants-13-00169-s001.zip › plants-2732671-supplementary.pdf]

Supplementary Materials

Comprehensive Characterization of Global Barley (*Hordeum vulgare* L.) Collection Using Agronomic Traits, β-Glucan Level, Phenolic Content, and Antioxidant Activities

Kebede Taye Desta [1,†], Yu-Mi Choi [1,†], Hyemyeong Yoon [1], Sukyeung Lee [2], Jungyoon Yi [1], Young-ah Jeon [1], Xiaohan Wang [1], Jin-Cheon Park [3], Kyeong-Min Kim [3] and Myoung-Jae Shin [1,*]

[1] National Agrobiodiversity Center, National Institute of Agricultural Sciences, Rural Development Administration, Jeonju 54874, Republic of Korea
[2] International Technology Cooperation Center, Technology Cooperation Bureau, Rural Development Administration, Jeonju 54875, Republic of Korea
[3] National Institute of Crop Science, Rural Development Administration, Wanju 55365, Republic of Korea
[*] Correspondence: smj1204@rda.go.kr
[†] These authors contributed equally to this work.

Table S1. The frequency of 367 barley accessions according to their origin.

Origin	Frequency (*n*)	Origin	Frequency (*n*)
AFG	11	KOR	64
ARG	5	LBN	3
CHE	60	MAR	11
CHL	1	MNG	12
CHN	14	NPL	14
DEU	1	PAK	11
ETH	54	PER	30
GEO	14	PRT	2
GRC	11	RUS	1
IND	14	TJK	2
IRN	4	TUR	14
ISR	6	UKR	1
ITA	1	UZB	4
JOR	2		
**Total (*n*= 367)**	**198**		**169**

AFG: Afghanistan, ARG: Argentia, CHE: Switzerland, CHL: Chile, CHN: China, DEU: Germany, ETH: Ethiopia, GEO: Georgia, GRC: Greece, IND: India, IRN: Iran, ISR: Israel, ITA: Italy, JOR: Jordan, KOR: Korea, LBN: Lebanon, MAR: Morocco, MNG: mongolia, NPL: Nepal, PAK: pakistan, PER: peru, PRT: portugal, RUS: Russia, TJK: Tajikistan, TUR: Turkey, UKR: Ukraine, UZB: Uzbekistan.

Table S2. Qualitative agro-morphological traits in global barely accessions grown in Korea.

Name	Origin	Qualitative agronomical traits						
		Growth habit	Erectness of flag leaf	Spike type	Spike erectness angle	Grain type	Cold damage	Lodging
Castelar-668	ARG	Erect	Erect	Six rows	Semi-erect	Hulled	Resistant	Nothing
GRA1015	UKR	Prostate	Semi-erect	Six rows	Semi-erect	Hulled	Resistant	Nothing
GRA1034	ITA	Prostate	Erect	Two rows	Horizontal	Hulled	Semi-resistant	Nothing
GRA2256	TJK	Intermediate	Semi-erect	Six rows	Erect	Hulled	Resistant	Nothing
GRA2621	GRC	Intermediate	Horizontal	Six rows	Semi-erect	Hulled	Semi-resistant	Nothing
GRA985	ARG	Intermediate	Semi-erect	Six rows	Erect	Hulled	Resistant	Nothing
UZB-BHJ-2002-12-3	UZB	Intermediate	Semi-erect	Six rows	Semi-erect	Hulled	Semi-resistant	Nothing
UZB-BHJ-2002-15-3	UZB	Intermediate	Semi-erect	Six rows	Semi-erect	Hulled	Resistant	Nothing
UZB-BHJ-2002-23-2	UZB	Intermediate	Semi-erect	Six rows	Semi-erect	Hulled	Semi-resistant	Nothing
IG 38956	ISR	Intermediate	Horizontal	Two rows	Drooping	Hulled	Intermediate	Nothing
IG 40039	JOR	Intermediate	Semi-erect	Two rows	Horizontal	Hulled	Semi-resistant	Nothing
B-205	PRT	Intermediate	Semi-erect	Six rows	Horizontal	Hulled	Nothing	Nothing
302	TUR	Intermediate	Semi-erect	Six rows	Semi-erect	Hulled	Resistant	Nothing
HVS 235	JOR	Prostate	Horizontal	Two rows	Horizontal	Hulled	Semi-resistant	Semi-resistant
HVS 355	LBN	Prostate	Horizontal	Two rows	Semi-drooping	Hulled	Semi-resistant	Nothing
HVS 366	LBN	Prostate	Horizontal	Two rows	Horizontal	Hulled	Resistant	Nothing
HVS 448-2	UZB	Prostate	Semi-erect	Two rows	Horizontal	Hulled	Resistant	Nothing
PI 202174	ARG	Prostate	Semi-erect	Two rows	Semi-erect	Hulled	Resistant	Nothing
PI 204705	TUR	Intermediate	Semi-erect	Two rows	Horizontal	Hulled	Semi-resistant	Nothing
PI 204875	TUR	Intermediate	Semi-erect	Two rows	Semi-erect	Hulled	Resistant	Nothing
PI 211049	AFG	Erect	Semi-erect	Two rows	Semi-erect	Hulled	Semi-resistant	Nothing

PI 220520	AFG	Intermediate	Semi-erect	Two rows	Semi-erect	Hulled	Resistant	Nothing
PI 223373	IRN	Intermediate	Semi-erect	Two rows	Erect	Hulled	Resistant	Nothing
PI 235639	DEU	Intermediate	Semi-erect	Two rows	Erect	Hulled	Resistant	Nothing
PI 244766	IRN	Intermediate	Semi-erect	Two rows	Erect	Hulled	Resistant	Nothing
PI 255142	CHL	Intermediate	Semi-erect	Two rows	Horizontal	Hulled	Semi-resistant	Nothing
PI 255161	ARG	Intermediate	Semi-erect	Two rows	Horizontal	Hulled	Resistant	Nothing
PI 282583	ISR	Intermediate	Horizontal	Two rows	Horizontal	Hulled	Semi-resistant	Nothing
PI 282649	ISR	Erect	Semi-erect	Two rows	Horizontal	Hulled	Semi-resistant	Nothing
C.P.I. 18967	ISR	Intermediate	Semi-erect	Two rows	Semi-erect	Hulled	Resistant	Nothing
Ischnatherum I	TJK	Prostate	Horizontal	Two rows	Horizontal	Hulled	Resistant	Nothing
Dinoor No. 949	ISR	Intermediate	Horizontal	Two rows	Horizontal	Hulled	Semi-resistant	Nothing
PI 304351	RUS	Intermediate	Semi-erect	Two rows	Horizontal	Hulled	Semi-resistant	Nothing
PI 304357	PRT	Intermediate	Semi-erect	Two rows	Semi-erect	Hulled	Semi-resistant	Nothing
HS 610	ISR	Intermediate	Horizontal	Two rows	Semi-erect	Hulled	Semi-resistant	Nothing
D-55	IRN	Intermediate	Semi-erect	Two rows	Semi-erect	Hulled	Nothing	Nothing
D-305	IRN	Intermediate	Horizontal	Two rows	Horizontal	Hulled	Semi-resistant	Nothing
PI 466252	LBN	Prostate	Horizontal	Two rows	Horizontal	Hulled	Resistant	Nothing
Castelar-034	ARG	Intermediate	Semi-erect	Two rows	Semi-erect	Hulless	Resistant	Nothing
CI10819	AFG	Erect	Erect	Six rows	Semi-erect	Hulled	Resistant	Resistant
Kwangsan 7-11	KOR	Erect	Semi-erect	Six rows	Horizontal	Hulless	Resistant	Nothing
Gonju 87-12	KOR	Erect	Erect	Six rows	Semi-erect	Hulled	Resistant	Nothing
Sancheong 87-34	KOR	Erect	Erect	Six rows	Semi-erect	Hulless	Resistant	Nothing
Sancheong 87-39	KOR	Intermediate	Horizontal	Six rows	Semi-erect	Hulled	Resistant	Nothing
Wolseong 87-133	KOR	Intermediate	Semi-erect	Six rows	Erect	Hulled	Resistant	Nothing
Gyeongju 87-19	KOR	Intermediate	Erect	Two rows	Semi-erect	Hulled	Resistant	Nothing
Gyeongju 87-46	KOR	Intermediate	Horizontal	Six rows	Erect	Hulled	Resistant	Nothing

Gochang 87-29	KOR	Intermediate	Erect	Six rows	Erect	Hulless	Resistant	Nothing
Suncheon 87-16	KOR	Intermediate	Semi-erect	Six rows	Semi-erect	Hulless	Resistant	Nothing
Jaeraebaekkwa	KOR	Erect	Erect	Six rows	Erect	Hulless	Resistant	Nothing
Gurye 89-11	KOR	Intermediate	Erect	Two rows	Drooping	Hulled	Resistant	Nothing
WIR1241	MNG	Erect	Erect	Six rows	Semi-drooping	Hulless	Semi-resistant	Nothing
WIR5598	MNG	Erect	Erect	Two rows	Erect	Hulless	Resistant	Nothing
WIR398	MNG	Intermediate	Horizontal	Six rows	Semi-drooping	Hulless	Resistant	Nothing
WIR1221	MNG	Intermediate	Semi-erect	Six rows	Horizontal	Hulless	Resistant	Resistant
WIR1089	MNG	Intermediate	Semi-erect	Six rows	Horizontal	Hulless	Intermediate	Intermediate
WIR92	MNG	Erect	Horizontal	Two rows	Semi-drooping	Hulless	Susceptible	Susceptible
WIR1531	MNG	Intermediate	Semi-erect	Six rows	Horizontal	Hulless	Intermediate	Intermediate
WIR1127	MNG	Erect	Horizontal	Six rows	Horizontal	Hulless	Semi-resistant	Semi-resistant
WIR4634	MNG	Intermediate	Semi-erect	Six rows	Semi-erect	Hulless	Semi-resistant	Semi-resistant
WIR1607	MNG	Erect	Semi-erect	Six rows	Semi-drooping	Hulled	Intermediate	Intermediate
Sua	CHN	Erect	Semi-erect	Six rows	Erect	Hulless	Semi-resistant	Semi-resistant
IG 26455	KOR	Erect	Horizontal	Six rows	Semi-drooping	Hulless	Resistant	Resistant
Pharona	IND	Erect	Semi-erect	Six rows	Semi-drooping	Hulless	Semi-resistant	Semi-resistant
Thangja 1	NPL	Intermediate	Erect	Six rows	Semi-erect	Hulless	Semi-resistant	Semi-resistant
TKN 24b	NPL	Intermediate	Erect	Six rows	Drooping	Hulled	Semi-resistant	Semi-resistant
CI 9970	ETH	Intermediate	Erect	Two rows	Semi-erect	Hulless	Resistant	Resistant
CI 6134	TUR	Prostate	Erect	Two rows	Erect	Hulled	Resistant	Resistant
CI 6221	TUR	Intermediate	Semi-erect	Two rows	Semi-erect	Hulless	Susceptible	Susceptible
CI 6222	TUR	Intermediate	Horizontal	Two rows	Semi-drooping	Hulless	Intermediate	Intermediate
Shirok Kapo	IND	Erect	Semi-erect	Six rows	Semi-drooping	Hulless	Severe	Nothing
Shirok	IND	Intermediate	Horizontal	Six rows	Semi-drooping	Hulless	Intermediate	Resistant
Abyssinian 13	ETH	Intermediate	Semi-erect	Two rows	Semi-erect	Hulless	Semi-resistant	Nothing

CIho 10381	ETH	Intermediate	Erect	Two rows	Semi-erect	Hulless	Semi-resistant	Nothing
K703	PAK	Erect	Erect	Six rows	Erect	Hulled	Semi-resistant	Nothing
PI 264912	GRC	Prostate	Erect	Six rows	Semi-erect	Hulled	Resistant	Nothing
Or Bishy Jo	PAK	Prostate	Horizontal	Six rows	Horizontal	Hulled	Resistant	Nothing
PI 270747	PER	Intermediate	Erect	Six rows	Semi-erect	Hulless	Semi-resistant	Nothing
H-2185	ETH	Erect	Semi-erect	Two rows	Horizontal	Hulless	Susceptible	Resistant
PI 316806	ETH	Erect	Semi-erect	Two rows	Erect	Hulless	Resistant	Nothing
PI 328305	TUR	Intermediate	Erect	Six rows	Erect	Hulled	Semi-resistant	Nothing
PI 328348	TUR	Intermediate	Semi-erect	Six rows	Semi-erect	Hulled	Semi-resistant	Nothing
PI 328362	TUR	Intermediate	Erect	Two rows	Horizontal	Hulled	Resistant	Nothing
PI 328414	GRC	Intermediate	Horizontal	Six rows	Semi-erect	Hulled	Semi-resistant	Resistant
PI 328420	GRC	Intermediate	Semi-erect	Six rows	Erect	Hulled	Semi-resistant	Nothing
PI 328425	GRC	Intermediate	Semi-erect	Six rows	Erect	Hulled	Semi-resistant	Nothing
PI 328427	GRC	Erect	Erect	Six rows	Erect	Hulled	Intermediate	Nothing
PI 328488	GRC	Intermediate	Semi-erect	Six rows	Erect	Hulled	Semi-resistant	Nothing
PI 328499	GRC	Intermediate	Erect	Six rows	Semi-erect	Hulled	Semi-resistant	Intermediate
PI 328508	GRC	Intermediate	Semi-erect	Six rows	Erect	Hulled	Semi-resistant	Nothing
PI 328521	GRC	Intermediate	Semi-erect	Six rows	Semi-erect	Hulled	Severe	Nothing
PI 328540	TUR	Prostate	Erect	Six rows	Semi-erect	Hulled	Resistant	Resistant
PI 328580	GRC	Intermediate	Erect	Six rows	Erect	Hulled	Intermediate	Nothing
PI 328884	CHE	Intermediate	Erect	Six rows	Erect	Hulled	Semi-resistant	Nothing
PI 328922	CHN	Intermediate	Horizontal	Two rows	Horizontal	Hulled	Semi-resistant	Nothing
PI 328978	ETH	Erect	Erect	Six rows	Erect	Hulled	Semi-resistant	Nothing
117b	ETH	Intermediate	Erect	Six rows	Semi-erect	Hulled	Semi-resistant	Nothing
PI 342215	TUR	Intermediate	Erect	Two rows	Horizontal	Hulled	Semi-resistant	Nothing
E 209/5	ETH	Erect	Erect	Two rows	Drooping	Hulled	Intermediate	Nothing

E 5/290	ETH	Erect	Erect	Two rows	Semi-erect	Hulled	Resistant	Nothing
E 364/4	ETH	Erect	Erect	Two rows	Semi-erect	Hulless	Resistant	Nothing
MOR 8/1	MAR	Intermediate	Erect	Two rows	Erect	Hulled	Semi-resistant	Nothing
MOR 8/2	MAR	Intermediate	Erect	Two rows	Erect	Hulled	Semi-resistant	Nothing
MOR 8/4	MAR	Intermediate	Erect	Two rows	Horizontal	Hulled	Resistant	Semi-resistant
E 38/5	ETH	Erect	Erect	Six rows	Semi-drooping	Hulled	Semi-resistant	Resistant
E 140/3	ETH	Erect	Erect	Six rows	Drooping	Hulled	Semi-resistant	Semi-resistant
E 272/2	ETH	Intermediate	Horizontal	Six rows	Semi-drooping	Hulled	Semi-resistant	Resistant
E 282/2	ETH	Intermediate	Erect	Six rows	Horizontal	Hulled	Semi-resistant	Nothing
E 350/7	ETH	Intermediate	Erect	Six rows	Erect	Hulled	Susceptible	Nothing
E 517/3	ETH	Intermediate	Semi-erect	Six rows	Semi-erect	Hulled	Severe	Nothing
E 545/4	ETH	Erect	Erect	Six rows	Drooping	Hulled	Intermediate	Nothing
E 549/3	ETH	Erect	Erect	Six rows	Semi-erect	Hulled	Intermediate	Nothing
E 560/6	ETH	Prostate	Erect	Six rows	Semi-erect	Hulled	Semi-resistant	Nothing
MOR 4/3	MAR	Erect	Semi-erect	Six rows	Erect	Hulled	Semi-resistant	Nothing
MOR 5/4	MAR	Erect	Erect	Six rows	Erect	Hulled	Semi-resistant	Nothing
MOR 5/6	MAR	Erect	Erect	Six rows	Erect	Hulled	Semi-resistant	Nothing
MOR 7/4	MAR	Intermediate	Semi-erect	Six rows	Semi-drooping	Hulled	Intermediate	Nothing
MOR 12/3	MAR	Intermediate	Erect	Six rows	Horizontal	Hulled	Intermediate	Nothing
MOR 12/9	MAR	Intermediate	Erect	Six rows	Erect	Hulled	Semi-resistant	Nothing
MOR 13/4	MAR	Intermediate	Erect	Six rows	Erect	Hulled	Resistant	Nothing
MOR 15/1	MAR	Intermediate	Erect	Six rows	Erect	Hulled	Semi-resistant	Nothing
27A	CHE	Erect	Semi-erect	Six rows	Semi-drooping	Hulless	Semi-resistant	Nothing
182A	CHE	Erect	Semi-erect	Two rows	Semi-erect	Hulled	Intermediate	Nothing
200G	CHE	Erect	Erect	Two rows	Semi-drooping	Hulled	Semi-resistant	Nothing
215A	CHE	Intermediate	Erect	Two rows	Semi-drooping	Hulled	Susceptible	Nothing

425A	CHE	Intermediate	Horizontal	Two rows	Drooping	Hulled	Semi-resistant	Nothing
445B	CHE	Intermediate	Horizontal	Two rows	Semi-drooping	Hulled	Semi-resistant	Nothing
446A	CHE	Intermediate	Horizontal	Two rows	Semi-drooping	Hulled	Resistant	Nothing
469A	CHE	Intermediate	Horizontal	Two rows	Semi-drooping	Hulled	Resistant	Nothing
480A	CHE	Intermediate	Semi-erect	Two rows	Horizontal	Hulled	Semi-resistant	Nothing
498A	CHE	Intermediate	Horizontal	Two rows	Horizontal	Hulled	Semi-resistant	Nothing
513A	CHE	Intermediate	Horizontal	Two rows	Drooping	Hulled	Semi-resistant	Nothing
523A	CHE	Erect	Horizontal	Two rows	Semi-drooping	Hulled	Resistant	Nothing
527IIC	CHE	Erect	Horizontal	Two rows	Semi-erect	Hulled	Intermediate	Nothing
530B	CHE	Erect	Horizontal	Two rows	Drooping	Hulled	Semi-resistant	Nothing
556B	CHE	Intermediate	Erect	Two rows	Semi-drooping	Hulled	Resistant	Nothing
557A	CHE	Erect	Horizontal	Two rows	Drooping	Hulled	Semi-resistant	Nothing
582A	CHE	Intermediate	Horizontal	Two rows	Drooping	Hulled	Resistant	Nothing
582C	CHE	Erect	Erect	Six rows	Semi-erect	Hulled	Resistant	Nothing
584B	CHE	Intermediate	Horizontal	Two rows	Horizontal	Hulled	Resistant	Nothing
589C	CHE	Intermediate	Semi-erect	Two rows	Semi-drooping	Hulled	Resistant	Nothing
590C	CHE	Intermediate	Horizontal	Two rows	Drooping	Hulled	Resistant	Nothing
781E	CHE	Erect	Horizontal	Two rows	Drooping	Hulled	Resistant	Nothing
864A	CHE	Intermediate	Erect	Two rows	Semi-erect	Hulled	Resistant	Nothing
959BA	CHE	Intermediate	Horizontal	Two rows	Drooping	Hulled	Resistant	Nothing
969A	CHE	Intermediate	Erect	Two rows	Horizontal	Hulled	Semi-resistant	Nothing
1080A	CHE	Erect	Horizontal	Two rows	Semi-drooping	Hulled	Resistant	Nothing
1247CB	CHE	Intermediate	Erect	Two rows	Semi-erect	Hulled	Resistant	Nothing
1317B	CHE	Intermediate	Semi-erect	Two rows	Drooping	Hulled	Resistant	Resistant
1352CD	CHE	Intermediate	Horizontal	Two rows	Semi-drooping	Hulled	Resistant	Nothing
1789BB	CHE	Intermediate	Erect	Two rows	Horizontal	Hulled	Resistant	Nothing

1820BA	CHE	Intermediate	Erect	Two rows	Horizontal	Hulled	Semi-resistant	Nothing
1857B	CHE	Intermediate	Horizontal	Two rows	Semi-drooping	Hulled	Resistant	Nothing
1851D	CHE	Intermediate	Horizontal	Two rows	Semi-drooping	Hulled	Resistant	Nothing
1879BA	CHE	Intermediate	Semi-erect	Two rows	Semi-drooping	Hulled	Resistant	Nothing
1884B	CHE	Intermediate	Semi-erect	Two rows	Semi-erect	Hulled	Semi-resistant	Nothing
1884F	CHE	Erect	Erect	Two rows	Horizontal	Hulled	Semi-resistant	Nothing
1900BA	CHE	Intermediate	Semi-erect	Two rows	Drooping	Hulled	Semi-resistant	Nothing
1948F	CHE	Intermediate	Erect	Two rows	Drooping	Hulled	Semi-resistant	Nothing
1964E	CHE	Intermediate	Semi-erect	Two rows	Semi-erect	Hulled	Resistant	Nothing
1968B	CHE	Intermediate	Semi-erect	Two rows	Semi-drooping	Hulled	Intermediate	Nothing
1981B	CHE	Intermediate	Erect	Two rows	Drooping	Hulled	Semi-resistant	Resistant
1986A	CHE	Intermediate	Semi-erect	Two rows	Drooping	Hulled	Resistant	Nothing
2003B	CHE	Intermediate	Erect	Two rows	Drooping	Hulled	Semi-resistant	Nothing
2028B	CHE	Erect	Horizontal	Two rows	Drooping	Hulled	Resistant	Nothing
2030B	CHE	Intermediate	Semi-erect	Two rows	Semi-drooping	Hulled	Semi-resistant	Nothing
2061B	CHE	Intermediate	Erect	Two rows	Drooping	Hulled	Semi-resistant	Nothing
2063B	CHE	Intermediate	Erect	Two rows	Drooping	Hulled	Resistant	Nothing
2073D	CHE	Intermediate	Erect	Two rows	Drooping	Hulled	Resistant	Nothing
2075B	CHE	Intermediate	Erect	Two rows	Semi-drooping	Hulled	Semi-resistant	Nothing
2085B	CHE	Intermediate	Semi-erect	Two rows	Semi-drooping	Hulled	Resistant	Nothing
2117C	CHE	Intermediate	Erect	Two rows	Drooping	Hulled	Resistant	Nothing
2123E	CHE	Intermediate	Erect	Two rows	Horizontal	Hulled	Intermediate	Nothing
2134D	CHE	Intermediate	Semi-erect	Six rows	Drooping	Hulless	Semi-resistant	Nothing
2155A	CHE	Intermediate	Semi-erect	Two rows	Erect	Hulled	Semi-resistant	Nothing
GAW 64-4	ETH	Erect	Erect	Two rows	Semi-drooping	Hulled	Intermediate	Nothing
GAW 72-11	ETH	Erect	Erect	Two rows	Semi-drooping	Hulled	Intermediate	Resistant

GAW 80-7	ETH	Erect	Erect	Two rows	Drooping	Hulled	Semi-resistant	Semi-resistant
GAW 89-11	ETH	Erect	Erect	Two rows	Drooping	Hulled	Semi-resistant	Semi-resistant
GAW 90-5	ETH	Erect	Erect	Two rows	Drooping	Hulled	Semi-resistant	Nothing
GAW 103-1	ETH	Intermediate	Semi-erect	Two rows	Horizontal	Hulless	Susceptible	Nothing
GAW 26-2	ETH	Erect	Semi-erect	Two rows	Horizontal	Hulled	Intermediate	Nothing
GAW 9-4	ETH	Erect	Erect	Six rows	Semi-erect	Hulled	Intermediate	Nothing
GAW 11-2	ETH	Erect	Erect	Six rows	Semi-erect	Hulled	Intermediate	Nothing
GAW 49-5	ETH	Intermediate	Semi-erect	Six rows	Erect	Hulled	Intermediate	Nothing
GAW 67-2	ETH	Erect	Erect	Six rows	Horizontal	Hulled	Semi-resistant	Nothing
GAW 69-8	ETH	Erect	Semi-erect	Six rows	Semi-drooping	Hulled	Semi-resistant	Nothing
GAW 70-6	ETH	Intermediate	Erect	Six rows	Semi-drooping	Hulled	Intermediate	Resistant
GAW 125-4	ETH	Erect	Semi-erect	Two rows	Semi-erect	Hulled	Susceptible	Nothing
GAW 129-2	ETH	Erect	Erect	Six rows	Semi-erect	Hulled	Semi-resistant	Nothing
GAW 130-1	ETH	Intermediate	Semi-erect	Six rows	Erect	Hulled	Intermediate	Nothing
GAW 130-2	ETH	Intermediate	Semi-erect	Six rows	Drooping	Hulled	Semi-resistant	Nothing
GAW 151-8	ETH	Erect	Semi-erect	Six rows	Erect	Hulled	Intermediate	Nothing
IAR/B/65-1	ETH	Intermediate	Erect	Two rows	Semi-erect	Hulled	Intermediate	Nothing
IAR/B/20-3	ETH	Erect	Semi-erect	Two rows	Semi-erect	Hulled	Susceptible	Nothing
IAR/B/22	ETH	Intermediate	Erect	Two rows	Drooping	Hulled	Intermediate	Nothing
IAR/B/160-1	ETH	Intermediate	Semi-erect	Two rows	Erect	Hulled	Intermediate	Nothing
IAR/B/161-1	ETH	Erect	Semi-erect	Six rows	Semi-erect	Hulled	Intermediate	Nothing
IAR/B/205-4	ETH	Erect	Semi-erect	Six rows	Semi-erect	Hulled	Intermediate	Nothing
IAR/B/411-3	ETH	Intermediate	Semi-erect	Six rows	Semi-erect	Hulled	Intermediate	Nothing
IAR/B/162-1	ETH	Erect	Erect	Six rows	Semi-erect	Hulled	Intermediate	Nothing
IAR/B/195-2	ETH	Intermediate	Erect	Six rows	Semi-erect	Hulled	Intermediate	Nothing
IAR/B/205-2	ETH	Erect	Erect	Six rows	Semi-drooping	Hulled	Semi-resistant	Resistant

N41	PAK	Intermediate	Erect	Six rows	Drooping	Hulless	Intermediate	Nothing
Gopal	IND	Intermediate	Semi-erect	Six rows	Erect	Hulled	Resistant	Nothing
C125	IND	Intermediate	Semi-erect	Six rows	Erect	Hulled	Susceptible	Nothing
N125	IND	Intermediate	Erect	Six rows	Erect	Hulless	Intermediate	Nothing
C145	IND	Erect	Semi-erect	Six rows	Horizontal	Hulled	Intermediate	Intermediate
UNA 8341	PER	Intermediate	Semi-erect	Six rows	Semi-erect	Hulless	Intermediate	Nothing
UNA 8387	PER	Intermediate	Semi-erect	Six rows	Semi-erect	Hulless	Intermediate	Resistant
UNA 8396	PER	Intermediate	Erect	Six rows	Semi-drooping	Hulless	Semi-resistant	Nothing
Qingki	CHN	Intermediate	Erect	Six rows	Drooping	Hulless	Semi-resistant	Nothing
PI 513174	PAK	Intermediate	Horizontal	Two rows	Semi-erect	Hulled	Resistant	Nothing
PI 513177	PAK	Intermediate	Erect	Two rows	Semi-erect	Hulless	Resistant	Nothing
PI 513225	PAK	Erect	Erect	Six rows	Horizontal	Hulless	Semi-resistant	Resistant
PI 513234	PAK	Erect	Semi-erect	Six rows	Drooping	Hulless	Intermediate	Resistant
PI 57100	GEO	Prostate	Erect	Six rows	Horizontal	Hulled	Resistant	Nothing
PRC92-27	CHN	Erect	Semi-erect	Six rows	Horizontal	Hulless	Semi-resistant	Nothing
WIR 19490	ETH	Intermediate	Horizontal	Two rows	Semi-drooping	Hulled	Resistant	Nothing
WIR 26598	ETH	Intermediate	Semi-erect	Two rows	Horizontal	Hulless	Resistant	Nothing
SN-26	GEO	Erect	Semi-erect	Two rows	Semi-drooping	Hulled	Intermediate	Nothing
SN-1065	GEO	Intermediate	Horizontal	Six rows	Drooping	Hulled	Semi-resistant	Resistant
SN-2131	GEO	Intermediate	Horizontal	Two rows	Drooping	Hulled	Semi-resistant	Nothing
SN-2158	GEO	Intermediate	Horizontal	Two rows	Drooping	Hulled	Semi-resistant	Semi-resistant
RNB-9	NPL	Intermediate	Semi-erect	Six rows	Semi-drooping	Hulless	Semi-resistant	Susceptible
RNB-10	NPL	Intermediate	Erect	Six rows	Semi-drooping	Hulless	Resistant	Susceptible
RNB-119	NPL	Erect	Semi-erect	Six rows	Drooping	Hulless	Intermediate	Resistant
RNB-132	NPL	Erect	Semi-erect	Six rows	Semi-drooping	Hulless	Intermediate	Nothing
RNB-141	NPL	Erect	Horizontal	Six rows	Drooping	Hulless	Intermediate	Nothing

RNB-169	NPL	Intermediate	Semi-erect	Six rows	Semi-erect	Hulless	Intermediate	Nothing
RNB-205	NPL	Erect	Semi-erect	Six rows	Horizontal	Hulless	Intermediate	Nothing
RNB-309	NPL	Intermediate	Semi-erect	Six rows	Semi-drooping	Hulless	Susceptible	Nothing
Nushera	PAK	Intermediate	Erect	Six rows	Semi-drooping	Hulled	Semi-resistant	Nothing
2825-1	PAK	Intermediate	Horizontal	Six rows	Semi-erect	Hulless	Semi-resistant	Nothing
PI 65208	CHN	Intermediate	Horizontal	Six rows	Semi-erect	Hulled	Semi-resistant	Intermediate
PI 69614	CHN	Intermediate	Semi-erect	Six rows	Drooping	Hulled	Semi-resistant	Nothing
Georgia	GEO	Intermediate	Horizontal	Two rows	Semi-drooping	Hulless	Intermediate	Nothing
SD 5067	KOR	Intermediate	Semi-erect	Six rows	Semi-erect	Hulless	Resistant	Nothing
GEO-MKH-2018-168	GEO	Prostate	Semi-erect	Six rows	Semi-drooping	Hulled	Semi-resistant	Nothing
K230774-2	CHE	Erect	Semi-erect	Six rows	Drooping	Hulless	Semi-resistant	Nothing
K235060-1	ETH	Intermediate	Semi-erect	Six rows	Erect	Hulless	Semi-resistant	Nothing
K242071-1	PER	Intermediate	Semi-erect	Six rows	Semi-drooping	Hulless	Intermediate	Nothing
K242071-2	PER	Intermediate	Semi-erect	Six rows	Semi-erect	Hulless	Semi-resistant	Nothing
K242142-2	PAK	Intermediate	Erect	Six rows	Drooping	Hulless	Intermediate	Intermediate
K242143-1	PAK	Intermediate	Semi-erect	Six rows	Semi-erect	Hulless	Intermediate	Resistant
K222037-2	TUR	Intermediate	Erect	Two rows	Horizontal	Hulled	Resistant	Nothing
KSL 180880	KOR	Intermediate	Erect	Six rows	Semi-erect	Hulled	Resistant	Nothing
KSL 180888	KOR	Intermediate	Semi-erect	Six rows	Semi-erect	Hulless	Semi-resistant	Nothing
KSL 180964	KOR	Intermediate	Semi-erect	Six rows	Erect	Hulled	Resistant	Nothing
GEO-HDY-2019-159	GEO	Prostate	Erect	Six rows	Erect	Hulled	Resistant	Nothing
Gyeongbukyeongcheon-2019-5	KOR	Intermediate	Semi-erect	Six rows	Horizontal	Hulled	Resistant	Nothing
KSL 191198	KOR	Intermediate	Semi-erect	Six rows	Horizontal	Hulled	Resistant	Nothing
Pimaek-3	KOR	Prostate	Horizontal	Six rows	Erect	Hulled	Semi-resistant	Nothing
Milyangjaeraejong 2ho	KOR	Intermediate	Erect	Six rows	Drooping	Hulled	Semi-resistant	Nothing
Oljujukha	KOR	Intermediate	Semi-erect	Six rows	Erect	Hulless	Semi-resistant	Nothing

Aeraemo	KOR	Intermediate	Semi-erect	Six rows	Erect	Hulless	Resistant	Nothing
Gyeongnamhamyang-1985-917	KOR	Intermediate	Semi-erect	Six rows	Semi-erect	Hulless	Resistant	Nothing
Gyeongbukchilgok-1985-962	KOR	Intermediate	Semi-erect	Six rows	Semi-erect	Hulless	Resistant	Nothing
Jeonbukiksan-1985-1094	KOR	Intermediate	Semi-erect	Six rows	Semi-erect	Hulless	Resistant	Nothing
Gyeonghihwaseong-1985-2801	KOR	Prostate	Semi-erect	Six rows	Semi-erect	Hulled	Resistant	Nothing
Gyeongnamtongyeong-1985-3201	KOR	Intermediate	Semi-erect	Six rows	Semi-erect	Hulless	Semi-resistant	Nothing
Jeonbukokgu-1985-4850	KOR	Intermediate	Semi-erect	Six rows	Semi-erect	Hulless	Resistant	Nothing
Gangwonmyeongju-1985-6585	KOR	Prostate	Semi-erect	Six rows	Horizontal	Hulled	Resistant	Nothing
Gangwonsamcheok-1985-6800	KOR	Prostate	Semi-erect	Six rows	Semi-erect	Hulled	Resistant	Nothing
Gangwonsamcheok-1985-6834	KOR	Prostate	Semi-erect	Six rows	Semi-erect	Hulled	Resistant	Nothing
Gangwonsamcheok-1985-6967	KOR	Prostate	Horizontal	Six rows	Erect	Hulled	Resistant	Nothing
Chungbukokcheon-1985-7311	KOR	Prostate	Semi-erect	Six rows	Erect	Hulled	Resistant	Nothing
Chungbukokcheon-1985-7350	KOR	Intermediate	Erect	Six rows	Semi-erect	Hulled	Resistant	Nothing
Chungnamseosan-1985-7579	KOR	Prostate	Semi-erect	Six rows	Drooping	Hulled	Resistant	Resistant
Chungnamseocheon-1985-7615	KOR	Intermediate	Semi-erect	Six rows	Semi-erect	Hulless	Resistant	Nothing
Chungnamseocheon-1985-7658	KOR	Intermediate	Semi-erect	Six rows	Erect	Hulled	Resistant	Nothing
Chungnamhongseong-1985-7726	KOR	Prostate	Semi-erect	Six rows	Erect	Hulled	Resistant	Nothing
Chungnamnonsan-1985-7810	KOR	Prostate	Semi-erect	Six rows	Drooping	Hulled	Resistant	Nothing
Chungnamnonsan-1985-7867	KOR	Prostate	Semi-erect	Six rows	Horizontal	Hulless	Nothing	Nothing
Chungnamnonsan-1985-7871	KOR	Prostate	Horizontal	Six rows	Drooping	Hulless	Resistant	Nothing
Chungnamnonsan-1985-8057	KOR	Prostate	Semi-erect	Six rows	Semi-drooping	Hulless	Resistant	Nothing
Chungnambuyeo-1985-8152	KOR	Intermediate	Semi-erect	Six rows	Semi-erect	Hulless	Resistant	Nothing
CI9980	ETH	Intermediate	Horizontal	Two rows	Drooping	Hulled	Resistant	Nothing
Jeonbukgochang-1985-13356	KOR	Intermediate	Semi-erect	Six rows	Semi-erect	Hulless	Resistant	Nothing
Jnju 87-9	KOR	Prostate	Semi-erect	Six rows	Semi-erect	Hulless	Resistant	Nothing
Sancheong 87-30	KOR	Prostate	Erect	Six rows	Semi-erect	Hulless	Resistant	Nothing

Jeonbukgimjae-1989-3410	KOR	Intermediate	Semi-erect	Six rows	Erect	Hulless	Resistant	Nothing
Chungbukokcheon-1995-437	KOR	Intermediate	Erect	Six rows	Semi-erect	Hulled	Resistant	Nothing
Gyeonghihwasseong-1995-440	KOR	Intermediate	Semi-erect	Six rows	Erect	Hulled	Semi-resistant	Nothing
Chungbukokcheon-1995-441	KOR	Intermediate	Erect	Six rows	Erect	Hulled	Semi-resistant	Nothing
Chungnamseosan-1995-445	KOR	Intermediate	Semi-erect	Six rows	Erect	Hulled	Semi-resistant	Nothing
Gyeonghihwasang-1995-447	KOR	Intermediate	Semi-erect	Six rows	Erect	Hulled	Semi-resistant	Nothing
Gyeonghipyeongtaek-1995-448	KOR	Intermediate	Semi-erect	Six rows	Erect	Hulled	Semi-resistant	Nothing
Gyeonghipyeongtaek-1995-449	KOR	Intermediate	Semi-erect	Six rows	Erect	Hulled	Resistant	Nothing
Chungbukokcheon-1995-450	KOR	Intermediate	Erect	Six rows	Erect	Hulled	Resistant	Nothing
Gyeonghiongjin-1995-451	KOR	Intermediate	Erect	Six rows	Erect	Hulled	Semi-resistant	Nothing
Gyeonghihwaseong-1995-453	KOR	Intermediate	Semi-erect	Six rows	Erect	Hulled	Semi-resistant	Nothing
Gangwonmyeongju-1995-457	KOR	Intermediate	Erect	Six rows	Erect	Hulled	Semi-resistant	Nothing
Geumseongjaerae	KOR	Intermediate	Semi-erect	Six rows	Semi-erect	Hulless	Semi-resistant	Nothing
Daegugoyangjaerae-2	KOR	Prostate	Erect	Six rows	Semi-drooping	Hulless	Resistant	Nothing
Jaeraeyukkag (A)	KOR	Intermediate	Semi-erect	Six rows	Semi-erect	Hulless	Resistant	Nothing
CI 6150	CHN	Prostate	Erect	Six rows	Drooping	Hulless	Resistant	Susceptible
Abyssinian 1139	ETH	Intermediate	Erect	Six rows	Drooping	Hulless	Semi-resistant	Resistant
CIho 3989-2	MNG	Intermediate	Erect	Six rows	Drooping	Hulled	Semi-resistant	Resistant
CIho 4169	AFG	Intermediate	Horizontal	Two rows	Semi-erect	Hulled	Semi-resistant	Semi-resistant
Wase Shu	KOR	Erect	Erect	Six rows	Semi-drooping	Hulless	Semi-resistant	Resistant
Jou Shirin abi	AFG	Intermediate	Horizontal	Six rows	Semi-erect	Hulled	Semi-resistant	Nothing
Si Leng Bai Da Mai	CHN	Intermediate	Semi-erect	Six rows	Semi-drooping	Hulled	Semi-resistant	Nothing
PI 270611	PER	Erect	Erect	Six rows	Semi-erect	Hulless	Semi-resistant	Nothing
PI 270633	PER	Intermediate	Horizontal	Six rows	Horizontal	Hulled	Semi-resistant	Nothing
PI 270637	PER	Intermediate	Horizontal	Six rows	Erect	Hulled	Semi-resistant	Nothing
PI 270675	PER	Intermediate	Horizontal	Six rows	Erect	Hulled	Semi-resistant	Nothing

PI 270721	PER	Erect	Horizontal	Six rows	Erect	Hulled	Resistant	Nothing
PI 270758	PER	Intermediate	Erect	Six rows	Horizontal	Hulled	Semi-resistant	Nothing
PI 328905	AFG	Intermediate	Horizontal	Two rows	Semi-erect	Hulled	Semi-resistant	Nothing
CI 9878	AFG	Erect	Erect	Six rows	Horizontal	Hulled	Semi-resistant	Intermediate
CIHo13250	ETH	Erect	Semi-erect	Two rows	Semi-erect	Hulless	Semi-resistant	Nothing
Tohoku Shiro Hadaka	CHN	Intermediate	Erect	Six rows	Erect	Hulless	Semi-resistant	Resistant
Shargundik 2	IND	Erect	Erect	Six rows	Semi-drooping	Hulless	Semi-resistant	Nothing
Jinan Dohadaka	KOR	Prostate	Semi-erect	Six rows	Semi-erect	Hulless	Resistant	Nothing
Buan Waessalbori	KOR	Erect	Semi-erect	Six rows	Semi-erect	Hulless	Semi-resistant	Nothing
CI 4136	AFG	Intermediate	Semi-erect	Six rows	Erect	Hulless	Intermediate	Nothing
CIho 3970-1	MNG	Erect	Erect	Six rows	Semi-erect	Hulless	Semi-resistant	Nothing
PI 176033	IND	Erect	Erect	Six rows	Drooping	Hulless	Semi-resistant	Nothing
Pangu	IND	Intermediate	Semi-erect	Six rows	Drooping	Hulless	Semi-resistant	Nothing
PI 176122	IND	Erect	Erect	Six rows	Drooping	Hulled	Resistant	Nothing
PI 202901	CHN	Erect	Erect	Six rows	Drooping	Hulless	Semi-resistant	Nothing
PI 270619	PER	Intermediate	Erect	Six rows	Drooping	Hulled	Semi-resistant	Nothing
PI 270647	PER	Intermediate	Horizontal	Six rows	Semi-erect	Hulled	Resistant	Nothing
PI 270683	PER	Intermediate	Semi-erect	Six rows	Semi-erect	Hulled	Semi-resistant	Nothing
PI 270704	PER	Intermediate	Semi-erect	Six rows	Erect	Hulled	Resistant	Nothing
PI 270749	PER	Intermediate	Horizontal	Six rows	Semi-drooping	Hulled	Resistant	Nothing
PI 270754	PER	Intermediate	Erect	Six rows	Semi-erect	Hulled	Resistant	Nothing
PI 270729	PER	Intermediate	Erect	Six rows	Semi-drooping	Hulless	Resistant	Nothing
1972A	CHE	Intermediate	Semi-erect	Six rows	Horizontal	Hulless	Resistant	Nothing
ST-96	CHN	Intermediate	Erect	Six rows	Drooping	Hulless	Resistant	Nothing
UNA 8461	PER	Erect	Semi-erect	Six rows	Drooping	Hulless	Semi-resistant	Nothing
PI 447319	CHN	Intermediate	Erect	Six rows	Semi-drooping	Hulless	Semi-resistant	Nothing

SN-6	GEO	Prostate	Semi-erect	Two rows	Semi-erect	Hulled	Resistant	Nothing
GEO-PHJ-2015-3-31	GEO	Prostate	Erect	Six rows	Semi-drooping	Hulled	Resistant	Resistant
PI 61572	GEO	Intermediate	Erect	Six rows	Drooping	Hulled	Resistant	Resistant
PI 61508	GEO	Intermediate	Erect	Two rows	Semi-drooping	Hulled	Semi-resistant	Nothing
PI 429990	IND	Erect	Semi-erect	Six rows	Drooping	Hulless	Semi-resistant	Nothing
PI 429610	NPL	Erect	Erect	Six rows	Erect	Hulless	Semi-resistant	Nothing
PI 427243	NPL	Erect	Erect	Six rows	Semi-erect	Hulless	Semi-resistant	Resistant
Cebada Blanca	PER	Intermediate	Horizontal	Six rows	Horizontal	Hulled	Resistant	Nothing
UNA 8338	PER	Erect	Erect	Six rows	Semi-erect	Hulless	Semi-resistant	Nothing
PI 270715	PER	Erect	Erect	Six rows	Semi-erect	Hulless	Resistant	Semi-resistant
CIho 4181	AFG	Intermediate	Semi-erect	Six rows	Horizontal	Hulless	Susceptible	Nothing
PI 370999	CHE	Intermediate	Horizontal	Two rows	Drooping	Hulled	Semi-resistant	Nothing
PI 371148	CHE	Intermediate	Horizontal	Two rows	Semi-drooping	Hulled	Resistant	Nothing
PI 573706	GEO	Intermediate	Horizontal	Two rows	Drooping	Hulled	Semi-resistant	Intermediate
PI 574091	NPL	Intermediate	Erect	Six rows	Semi-drooping	Hulless	Intermediate	Nothing
PI 477805	PER	Erect	Erect	Six rows	Semi-erect	Hulless	Semi-resistant	Semi-resistant
PI 477851	PER	Erect	Erect	Six rows	Semi-drooping	Hulless	Semi-resistant	Semi-resistant
PI 510561	PER	Intermediate	Erect	Six rows	Horizontal	Hulless	Susceptible	Nothing
PI 342163	TUR	Intermediate	Erect	Two rows	Semi-erect	Hulled	Semi-resistant	Nothing
CIho 6962	AFG	Erect	Erect	Six rows	Erect	Hulless	Intermediate	Nothing
PI 270707	PER	Erect	Erect	Six rows	Horizontal	Hulless	Semi-resistant	Nothing
PI 296470	ETH	Intermediate	Semi-erect	Two rows	Horizontal	Hulless	Intermediate	Nothing
PI 328189	TUR	Prostate	Semi-erect	Six rows	Erect	Hulled	Resistant	Nothing
PI 510558	PER	Erect	Erect	Six rows	Semi-erect	Hulless	Semi-resistant	Intermediate
PI 559514	NPL	Erect	Semi-erect	Six rows	Drooping	Hulless	Semi-resistant	Susceptible
OUI 426	IND	Intermediate	Erect	Six rows	Semi-erect	Hulless	Semi-resistant	Resistant

PI 270618	PER	Intermediate	Erect	Six rows	Semi-drooping	Hulless	Intermediate	Semi-resistant
PI 27829	GEO	Prostate	Semi-erect	Six rows	Horizontal	Hulled	Semi-resistant	Nothing
CIho 9940	CHN	Erect	Erect	Six rows	Drooping	Hulless	Intermediate	Semi-resistant
PI 477832	PER	Erect	Erect	Six rows	Semi-erect	Hulless	Intermediate	Resistant
PI 366428	AFG	Intermediate	Semi-erect	Two rows	Semi-drooping	Hulled	Semi-resistant	Resistant
PI 69607	CHN	Intermediate	Erect	Six rows	Drooping	Hulled	Semi-resistant	Intermediate
OUI 420	IND	Erect	Semi-erect	Six rows	Horizontal	Hulless	Intermediate	Susceptible
PI 370970	CHE	Intermediate	Erect	Six rows	Drooping	Hulless	Semi-resistant	Nothing
Betaone	KOR	Intermediate	Semi-erect	Six rows	Erect	Hulless	Resistant	Nothing
Saechalssalbori	KOR	Erect	Erect	Six rows	Erect	Hulless	Semi-resistant	Nothing
Heuknuri	KOR	Erect	Semi-erect	Six rows	Semi-erect	Hulless	Resistant	Nothing
Heukdahyang	KOR	Erect	Semi-erect	Six rows	Semi-erect	Hulled	Resistant	Nothing
Saessalbori	KOR	Intermediate	Semi-erect	Six rows	Semi-erect	Hulless	Resistant	Nothing

Coutry codes representations are similar to those shown in Table S1 foot notes.

Table S3. Quantitative agronomical traits and biochemical contents in global barely accessions grown in Korea.

Name	Origin	Average values of agronomical traits								Average values of biochemical traits				
		DH	DM	DHM	CL	SL	AL	GPP	TGW	β-GL	TPC	ABTS	DPPH	RP
Castelar-668	ARG	204	234	30	70.07	13.17	2.20	41.20	3.00	0.14	4.07	8.60	2.68	3.60
GRA1015	UKR	198	233	35	109.07	13.63	4.13	20.80	10.40	0.23	4.14	8.92	3.32	4.30
GRA1034	ITA	187	221	34	82.17	7.73	4.43	14.80	47.70	5.25	2.95	6.58	2.89	3.30
GRA2256	TJK	201	229	28	108.67	14.40	5.03	14.00	12.00	0.35	4.85	10.65	4.20	6.13
GRA2621	GRC	201	229	28	115.17	13.03	5.60	16.80	11.00	0.43	6.22	13.54	6.24	7.86
GRA985	ARG	195	235	40	62.33	9.67	1.80	41.60	3.00	0.15	3.88	8.33	2.66	3.60
UZB-BHJ-2002-12-3	UZB	189	221	32	35.33	7.17	3.93	14.00	10.40	0.52	4.16	8.32	3.42	3.50
UZB-BHJ-2002-15-3	UZB	182	218	36	37.00	5.57	3.43	21.60	5.00	0.56	3.31	6.99	2.54	3.25
UZB-BHJ-2002-23-2	UZB	189	221	32	32.23	7.77	4.17	14.80	11.70	0.66	4.39	9.93	3.81	3.82
IG 38956	ISR	187	217	30	77.57	9.67	17.53	14.80	40.00	7.08	3.53	8.76	3.30	3.24
IG 40039	JOR	173	213	40	64.17	8.83	14.83	13.20	37.40	5.38	3.40	7.88	2.60	2.80
B-205	PRT	203	234	31	23.23	5.57	1.87	23.20	5.40	0.44	3.78	8.56	2.95	3.36
302	TUR	213	238	25	23.50	6.33	2.33	27.20	3.00	0.33	3.60	7.68	2.43	2.58
HVS 235	JOR	169	210	41	76.67	8.37	10.47	13.20	41.00	5.49	3.07	7.28	2.70	2.74
HVS 355	LBN	182	214	32	87.67	10.07	21.30	10.80	48.00	5.02	3.00	7.71	2.75	2.53
HVS 366	LBN	182	213	31	81.37	10.57	20.37	11.60	45.70	5.89	3.25	7.06	2.85	2.81
HVS 448-2	UZB	180	214	34	91.17	8.33	14.90	14.80	38.00	5.22	3.59	8.11	3.11	3.23
PI 202174	ARG	183	219	36	53.40	7.77	5.97	20.00	6.00	0.46	3.64	8.77	2.55	2.49
PI 204705	TUR	194	224	30	53.23	9.60	4.33	18.80	10.70	0.56	4.10	8.86	3.19	2.62
PI 204875	TUR	202	234	32	95.40	13.17	4.77	25.00	9.00	0.30	4.37	9.83	3.85	4.05
PI 211049	AFG	191	224	33	34.90	8.83	4.37	19.20	10.70	0.46	3.49	7.43	2.94	2.71
PI 220520	AFG	182	214	32	38.83	6.33	4.33	21.60	4.00	0.56	3.30	6.02	2.22	2.68
PI 223373	IRN	180	215	35	54.83	6.63	3.10	26.40	4.00	0.61	3.89	6.91	3.17	3.68

PI 235639	DEU	180	215	35	79.00	9.03	16.20	12.40	34.70	7.67	3.21	6.48	3.15	3.67
PI 244766	IRN	192	227	35	33.57	7.07	3.90	15.20	4.40	0.51	3.30	6.51	2.53	2.82
PI 255142	CHL	192	229	37	25.50	6.47	4.07	14.80	4.00	0.69	3.53	6.98	2.87	3.14
PI 255161	ARG	191	224	33	32.00	6.70	3.40	19.60	4.00	0.71	3.63	7.18	3.04	3.20
PI 282583	ISR	180	214	34	82.10	9.10	17.80	10.80	45.00	5.32	3.42	6.37	2.83	3.26
PI 282649	ISR	182	214	32	75.20	7.30	18.30	16.40	63.70	5.70	2.67	5.32	2.32	2.51
C.P.I. 18967	ISR	174	216	42	38.13	5.10	3.80	12.80	4.40	0.81	3.73	7.02	3.03	3.13
Ischnatherum I	TJK	194	219	25	81.40	10.50	19.63	15.60	47.70	4.89	3.14	6.68	2.89	3.13
Dinoor No. 949	ISR	178	212	34	78.57	10.17	19.33	10.40	42.70	6.58	3.55	7.31	3.16	3.34
PI 304351	RUS	196	233	37	37.50	7.27	4.60	26.40	9.00	0.60	3.94	5.86	3.02	2.94
PI 304357	PRT	201	238	37	37.00	9.13	6.07	18.80	8.40	0.59	4.51	6.95	3.69	3.39
HS 610	ISR	180	215	35	92.83	11.20	19.40	21.60	48.70	5.46	3.15	4.80	2.47	2.56
D-55	IRN	187	218	31	29.43	4.93	1.60	16.00	6.40	0.47	3.15	5.31	2.60	2.75
D-305	IRN	178	214	36	84.17	9.53	16.03	14.80	39.00	6.85	3.15	5.07	2.57	2.60
PI 466252	LBN	177	212	35	91.93	11.40	20.13	18.00	37.70	6.10	3.22	5.22	2.59	2.57
Castelar-034	ARG	205	238	33	64.83	8.33	0.93	40.80	3.40	0.43	4.09	5.70	1.93	1.95
CI10819	AFG	182	210	28	78.33	7.00	9.10	56.00	48.70	2.99	2.81	4.98	2.53	2.54
Kwangsan 7-11	KOR	175	210	35	60.50	5.00	4.83	61.80	40.70	3.55	3.13	5.51	2.42	2.26
Gonju 87-12	KOR	175	207	32	71.17	4.17	3.63	43.80	41.00	3.14	3.06	5.19	2.65	2.60
Sancheong 87-34	KOR	173	206	33	78.67	6.20	10.87	46.80	33.40	3.85	3.28	5.81	2.79	2.70
Sancheong 87-39	KOR	180	212	32	78.47	4.33	4.93	40.80	36.40	3.31	2.96	5.75	2.69	2.61
Wolseong 87-133	KOR	187	213	26	83.10	3.83	3.73	42.40	39.00	3.48	3.13	6.03	2.80	2.60
Gyeongju 87-19	KOR	180	211	31	81.50	8.33	13.17	24.40	51.70	3.47	3.58	6.03	2.31	2.20
Gyeongju 87-46	KOR	179	211	32	75.50	4.80	10.40	54.80	37.40	4.33	3.98	6.48	3.01	3.05
Gochang 87-29	KOR	173	206	33	75.37	5.93	10.53	62.00	32.70	3.72	3.28	6.06	2.97	2.73
Suncheon 87-16	KOR	174	208	34	60.33	4.33	5.27	56.80	32.70	3.51	4.30	7.35	3.58	3.28

Jaeraebaekkwa	KOR	178	210	32	82.17	5.20	4.17	56.40	34.70	3.03	3.29	6.21	2.92	2.55
Gurye 89-11	KOR	179	208	29	89.50	9.30	14.47	27.60	47.70	3.10	3.76	6.40	2.68	2.30
WIR1241	MNG	175	208	33	85.43	6.80	7.63	49.60	45.70	4.28	2.89	5.24	2.92	2.86
WIR5598	MNG	180	212	32	62.67	5.30	12.20	18.60	52.40	4.45	2.81	5.27	2.73	2.71
WIR398	MNG	183	216	33	65.00	8.13	12.97	61.60	37.40	3.95	3.08	5.56	3.10	3.16
WIR1221	MNG	188	216	28	75.53	6.23	13.10	44.60	39.40	3.95	2.24	3.92	2.17	2.08
WIR1089	MNG	194	223	29	60.17	6.93	0.00	38.60	45.00	3.40	2.63	4.64	2.30	2.37
WIR92	MNG	184	220	36	57.90	7.07	17.20	19.80	60.00	4.71	2.49	4.67	2.23	2.06
WIR1531	MNG	212	228	16	71.20	4.10	11.60	43.00	36.70	4.28	2.60	5.54	2.65	2.46
WIR1127	MNG	177	207	30	84.67	6.27	8.07	50.80	49.40	4.59	3.24	5.07	2.87	2.85
WIR4634	MNG	187	221	34	62.83	3.50	11.13	47.20	46.40	4.06	2.34	3.82	2.09	2.00
WIR1607	MNG	170	211	41	46.10	4.40	9.83	48.40	43.70	3.43	3.27	5.42	2.63	2.54
Sua	CHN	187	222	35	62.83	6.00	7.40	42.60	52.40	5.39	2.71	4.28	2.06	1.80
IG 26455	KOR	176	213	37	85.37	8.83	14.83	59.60	35.00	3.35	3.43	6.29	3.23	3.17
Pharona	IND	174	210	36	50.73	7.73	12.00	42.60	47.70	4.54	2.55	5.20	2.94	2.72
Thangja 1	NPL	179	214	35	74.80	5.53	10.90	45.80	39.70	4.88	2.98	6.23	3.29	2.93
TKN 24b	NPL	173	210	37	85.93	7.10	7.50	37.60	37.00	3.32	2.87	5.57	3.05	2.87
CI 9970	ETH	180	208	28	70.07	8.60	15.17	20.80	48.70	4.25	2.48	4.86	2.50	2.21
CI 6134	TUR	191	217	26	76.43	8.57	13.80	26.60	60.40	3.17	2.73	5.65	2.97	2.67
CI 6221	TUR	189	222	33	82.57	11.17	19.77	22.60	63.40	4.31	2.58	5.33	2.52	2.14
CI 6222	TUR	184	223	39	77.50	12.00	21.30	23.20	59.00	4.01	2.60	5.62	2.64	2.11
Shirok Kapo	IND	199	229	30	73.97	7.07	13.87	29.00	65.00	4.76	2.42	4.21	2.02	1.93
Shirok	IND	180	222	42	66.50	8.10	11.53	50.40	60.00	4.57	2.39	4.05	1.93	1.72
Abyssinian 13	ETH	188	221	33	70.00	6.80	15.77	25.00	59.40	5.26	3.67	6.27	3.02	2.56
CIho 10381	ETH	180	206	26	66.33	7.93	19.37	18.60	48.40	4.87	2.59	5.29	2.50	2.13
K703	PAK	174	210	36	81.33	4.50	6.27	36.00	45.70	3.33	2.35	4.87	2.68	2.53

PI 264912	GRC	187	216	29	90.83	7.30	12.50	38.40	48.00	3.66	2.74	5.97	3.39	2.99
Or Bishy Jo	PAK	180	222	42	82.57	7.67	12.67	47.00	47.70	3.08	2.55	5.31	2.74	2.48
PI 270747	PER	191	218	27	71.17	7.20	0.00	52.80	43.40	6.15	2.69	5.37	2.85	2.52
H-2185	ETH	180	210	30	65.33	8.40	16.77	18.60	62.00	4.95	2.57	5.30	2.85	2.50
PI 316806	ETH	176	205	29	79.50	5.80	14.17	24.40	56.70	4.24	2.01	4.14	2.05	1.89
PI 328305	TUR	177	210	33	53.50	3.70	10.23	31.20	55.40	3.51	2.75	5.96	3.09	2.64
PI 328348	TUR	197	229	32	60.93	4.57	12.73	37.20	46.70	3.65	3.07	5.25	2.71	2.50
PI 328362	TUR	180	212	32	64.90	8.23	13.90	20.40	55.70	4.31	2.50	5.28	2.79	2.49
PI 328414	GRC	175	213	38	67.67	6.23	11.43	48.40	56.00	3.92	2.91	5.88	3.07	2.65
PI 328420	GRC	191	222	31	86.17	6.73	13.90	40.80	50.70	3.19	3.51	6.98	3.70	3.34
PI 328425	GRC	180	210	30	56.90	5.73	11.50	26.80	49.40	2.83	3.16	6.63	3.08	2.81
PI 328427	GRC	187	216	29	64.27	4.83	11.23	32.20	55.40	3.41	2.95	4.97	2.69	2.68
PI 328488	GRC	182	215	33	74.83	4.17	18.23	44.00	62.70	3.24	2.90	4.67	2.53	2.50
PI 328499	GRC	169	204	35	76.50	5.33	10.50	42.60	55.00	3.25	3.16	5.27	2.69	2.67
PI 328508	GRC	185	216	30	83.17	6.17	16.30	44.80	60.00	2.57	2.83	4.58	2.51	2.50
PI 328521	GRC	194	223	29	83.17	4.73	14.27	45.00	62.40	3.12	2.74	4.28	2.12	2.15
PI 328540	TUR	180	210	30	79.00	5.87	9.40	47.60	54.00	4.19	2.55	4.84	2.38	2.33
PI 328580	GRC	176	211	35	76.50	4.33	13.67	29.20	53.00	3.84	3.43	6.90	3.43	3.16
PI 328884	CHE	185	220	35	71.13	4.17	11.77	37.80	55.40	3.83	2.71	4.35	2.19	2.17
PI 328922	CHN	179	213	34	69.13	7.03	13.67	20.00	57.70	3.77	2.87	4.58	2.16	2.05
PI 328978	ETH	180	213	33	79.80	5.23	13.00	38.80	46.40	3.48	2.64	4.62	2.42	2.21
117b	ETH	188	216	28	71.17	3.77	9.17	43.80	46.40	3.54	3.25	5.72	2.94	2.73
PI 342215	TUR	181	213	32	58.93	7.53	11.93	19.00	53.00	3.46	2.75	5.64	2.60	2.39
E 209/5	ETH	177	207	30	75.53	8.07	17.27	13.60	55.70	3.79	2.88	4.81	2.60	2.69
E 5/290	ETH	185	215	30	79.43	6.33	15.53	23.60	56.40	4.40	2.97	4.86	2.64	2.59
E 364/4	ETH	179	209	30	70.67	9.00	16.90	18.00	54.40	4.97	2.43	4.45	2.28	2.24

MOR 8/1	MAR	174	210	36	46.17	5.77	11.30	16.20	61.00	4.18	2.52	5.07	2.44	2.34
MOR 8/2	MAR	171	206	35	52.33	5.17	12.67	17.80	57.70	3.83	2.52	4.98	2.47	2.31
MOR 8/4	MAR	171	205	34	56.83	6.17	9.50	16.80	60.70	3.86	2.55	5.20	2.57	2.36
E 38/5	ETH	176	208	32	85.17	6.23	11.60	40.60	46.00	3.54	2.65	5.75	2.78	2.33
E 140/3	ETH	173	207	34	73.33	7.33	7.73	46.00	39.00	3.28	2.82	4.54	2.55	2.42
E 272/2	ETH	178	210	32	90.83	7.43	11.33	67.40	43.70	3.13	2.70	4.66	2.57	2.36
E 282/2	ETH	180	215	35	83.73	7.80	11.27	64.20	44.70	3.95	2.79	4.72	2.74	2.41
E 350/7	ETH	185	215	30	74.17	4.73	11.60	38.00	50.00	3.67	2.93	4.90	2.46	2.31
E 517/3	ETH	182	227	45	69.83	6.93	13.43	29.00	55.00	4.26	3.61	4.59	2.24	2.36
E 545/4	ETH	181	212	31	59.67	5.83	10.10	25.40	44.00	3.64	3.00	4.62	2.28	2.47
E 549/3	ETH	191	222	31	58.33	4.53	10.50	23.00	50.40	4.10	3.52	4.80	2.24	2.39
E 560/6	ETH	201	227	26	52.93	3.80	9.03	31.80	45.00	3.22	2.82	4.59	2.44	2.48
MOR 4/3	MAR	182	213	31	57.93	5.50	11.70	39.80	56.70	4.06	3.11	4.65	2.38	2.54
MOR 5/4	MAR	182	215	33	64.77	5.57	15.77	37.60	59.40	3.45	2.99	4.43	2.13	2.21
MOR 5/6	MAR	181	215	34	68.00	6.30	14.47	33.20	61.00	3.62	3.04	4.92	2.26	2.25
MOR 7/4	MAR	173	209	36	56.00	4.83	17.10	30.40	48.70	3.84	3.51	5.09	2.53	2.70
MOR 12/3	MAR	180	211	31	67.67	7.00	13.67	27.80	45.00	3.15	3.31	5.14	2.64	2.70
MOR 12/9	MAR	182	214	32	82.57	7.17	16.17	47.20	61.00	3.83	2.89	4.17	2.17	1.98
MOR 13/4	MAR	182	213	31	67.53	7.30	13.57	36.40	62.00	3.91	2.68	4.35	2.13	2.05
MOR 15/1	MAR	189	216	27	62.40	5.13	12.33	29.00	46.70	2.87	3.47	5.83	2.99	2.86
27A	CHE	180	211	31	76.33	6.00	9.40	46.40	34.40	3.93	3.44	5.92	3.20	3.25
182A	CHE	192	224	32	53.30	5.73	13.07	12.80	52.40	4.06	3.11	5.35	2.71	2.74
200G	CHE	190	217	27	62.83	7.20	14.97	23.20	45.00	3.64	3.35	5.57	2.91	2.90
215A	CHE	201	223	22	66.83	10.13	15.60	21.60	49.00	4.13	3.06	5.11	2.31	2.37
425A	CHE	182	214	32	66.00	7.30	14.73	23.40	44.40	3.49	2.87	5.54	2.80	2.60
445B	CHE	187	216	29	60.53	8.63	12.77	24.20	45.40	3.69	3.06	5.80	2.94	2.90

446A	CHE	185	216	31	74.43	9.43	15.33	26.40	46.70	3.65	3.25	5.51	2.81	2.87
469A	CHE	182	216	34	75.33	9.23	14.90	24.20	48.70	3.49	3.10	5.57	2.79	2.84
480A	CHE	185	215	30	75.50	9.03	13.30	26.00	47.00	3.22	2.83	4.58	2.37	2.27
498A	CHE	183	214	31	73.03	6.87	13.80	24.40	45.00	3.45	3.15	5.28	2.62	2.61
513A	CHE	179	215	36	74.00	6.87	12.03	16.20	46.70	3.05	3.22	5.59	2.84	2.80
523A	CHE	182	215	33	66.50	7.63	15.20	25.00	45.40	3.61	3.01	4.20	2.52	2.70
527IIC	CHE	191	216	25	72.27	8.97	15.27	18.40	48.00	3.51	3.38	4.80	2.78	3.02
530B	CHE	187	215	28	70.93	7.13	13.53	22.60	47.00	3.52	3.41	4.42	2.66	2.79
556B	CHE	187	216	29	73.50	7.17	13.20	24.60	43.70	3.37	2.92	4.33	2.46	2.58
557A	CHE	185	215	30	72.77	8.00	12.30	18.00	46.70	3.23	3.20	4.58	2.62	2.89
582A	CHE	182	214	32	76.93	7.17	13.90	24.00	43.00	4.16	3.35	4.85	2.61	2.70
582C	CHE	182	213	31	81.83	6.07	7.30	48.00	44.40	3.92	3.31	4.95	2.54	2.70
584B	CHE	182	214	32	81.17	7.87	13.97	23.20	46.70	3.76	3.82	4.95	2.88	3.00
589C	CHE	181	214	33	76.63	7.27	10.77	22.00	45.00	3.10	3.21	4.48	2.73	2.89
590C	CHE	180	210	30	92.17	10.17	10.67	27.40	47.70	3.24	3.30	4.64	2.65	2.75
781E	CHE	182	211	29	93.17	7.60	11.93	20.20	48.40	2.85	3.47	5.14	2.75	2.94
864A	CHE	185	216	30	72.77	6.97	13.20	20.80	51.40	3.29	3.85	5.34	2.73	2.88
959BA	CHE	177	207	30	83.67	9.70	12.67	25.80	52.00	3.34	2.32	4.80	2.74	2.88
969A	CHE	177	211	34	78.60	9.17	12.73	23.00	49.70	2.89	2.29	4.73	2.62	2.75
1080A	CHE	180	213	33	74.80	9.43	14.93	25.60	45.00	3.22	2.48	4.67	2.77	2.86
1247CB	CHE	190	216	26	68.77	8.53	13.40	21.60	57.70	3.39	2.21	4.64	2.76	2.82
1317B	CHE	176	206	30	89.40	9.07	14.00	24.40	48.40	2.97	2.26	4.74	2.72	2.81
1352CD	CHE	185	215	30	86.27	9.43	17.77	26.00	54.00	3.19	2.50	4.85	2.63	2.81
1789BB	CHE	191	215	24	81.67	8.07	11.60	21.80	51.40	3.21	2.34	4.61	2.37	2.45
1820BA	CHE	191	215	24	83.00	9.13	14.60	18.60	52.40	3.42	2.83	4.35	2.59	2.75
1857B	CHE	188	215	27	86.03	9.17	16.27	24.20	50.70	3.73	2.50	4.09	2.54	2.64

1851D	CHE	188	215	27	82.00	8.53	12.77	23.80	47.70	3.84	2.61	4.68	2.80	2.83
1879BA	CHE	191	215	24	77.57	9.17	15.97	24.00	48.70	3.76	2.44	4.37	2.53	2.55
1884B	CHE	191	215	24	68.33	7.27	14.83	25.20	48.70	3.89	2.81	5.46	3.06	3.07
1884F	CHE	190	215	25	76.17	8.77	13.33	24.60	47.40	3.52	2.21	4.74	2.63	2.50
1900BA	CHE	190	215	25	82.43	8.63	14.17	24.40	45.70	4.26	2.99	5.93	3.22	2.93
1948F	CHE	191	214	23	67.80	8.90	15.13	18.20	44.00	3.40	2.98	6.02	3.21	3.00
1964E	CHE	190	214	24	83.40	10.93	15.07	24.80	49.40	3.39	2.79	6.25	3.40	3.14
1968B	CHE	201	223	22	74.50	9.73	14.17	21.60	51.00	3.68	2.88	5.13	2.76	2.48
1981B	CHE	179	212	33	95.67	11.23	14.33	28.40	48.00	3.52	2.35	5.10	2.65	2.35
1986A	CHE	175	206	31	94.30	10.17	13.70	24.40	43.40	3.24	2.53	5.78	2.96	2.68
2003B	CHE	181	212	31	91.83	10.33	14.30	21.60	49.70	3.45	2.55	4.71	2.63	2.62
2028B	CHE	183	212	29	86.83	9.67	14.53	29.20	44.40	3.50	2.56	5.77	3.00	2.79
2030B	CHE	196	219	23	82.57	10.43	16.27	23.60	44.70	3.64	2.80	5.06	2.75	2.44
2061B	CHE	177	211	34	86.00	9.93	14.57	26.20	52.00	3.08	2.09	4.34	2.09	1.81
2063B	CHE	175	206	31	81.17	9.00	12.00	23.80	48.70	3.11	2.47	5.65	2.73	2.47
2073D	CHE	184	212	28	91.13	7.90	12.43	21.80	45.00	3.63	2.24	5.26	2.97	2.80
2075B	CHE	187	214	27	93.50	9.67	14.17	20.60	48.00	2.96	2.31	5.14	2.84	2.62
2085B	CHE	181	211	30	91.50	8.90	14.03	25.00	43.70	4.17	2.33	5.42	3.01	2.74
2117C	CHE	181	210	29	89.00	9.13	13.33	26.80	46.40	3.48	2.28	5.54	2.93	2.73
2123E	CHE	188	215	27	90.50	7.57	12.13	21.00	50.40	3.90	2.45	5.80	3.25	2.96
2134D	CHE	178	206	28	89.67	7.20	8.33	49.60	34.40	3.03	2.59	6.22	3.40	3.04
2155A	CHE	181	214	33	74.50	6.50	0.00	51.40	49.40	3.35	2.23	5.32	2.68	2.45
GAW 64-4	ETH	180	213	33	81.50	10.23	18.33	22.40	62.70	4.51	2.27	4.05	1.91	1.95
GAW 72-11	ETH	179	213	34	82.17	9.77	14.87	23.20	68.00	4.16	1.79	3.27	1.72	1.63
GAW 80-7	ETH	175	206	31	78.00	8.00	13.67	20.60	55.00	4.64	2.19	4.07	2.24	2.00
GAW 89-11	ETH	173	205	32	70.00	8.67	14.33	39.00	55.70	4.10	2.19	4.52	2.26	2.12

GAW 90-5	ETH	169	205	36	68.83	7.10	13.17	24.20	47.00	3.59	2.69	5.99	3.05	2.90
GAW 103-1	ETH	187	216	29	61.07	7.20	17.60	21.00	54.40	3.87	2.60	3.97	1.96	1.84
GAW 26-2	ETH	188	217	29	107.13	8.60	17.13	18.20	66.00	3.62	3.18	4.88	2.20	2.18
GAW 9-4	ETH	185	216	30	87.67	9.27	18.17	38.80	52.40	2.81	2.56	4.73	2.35	2.26
GAW 11-2	ETH	200	228	28	73.17	7.67	16.50	42.40	49.40	3.54	2.20	3.95	1.84	1.73
GAW 49-5	ETH	191	218	27	87.10	6.10	12.20	45.60	52.70	3.45	2.57	4.83	2.42	2.33
GAW 67-2	ETH	174	209	35	80.60	5.57	9.80	46.20	44.00	3.89	2.86	5.68	2.73	2.60
GAW 69-8	ETH	171	208	37	61.50	5.33	7.67	27.20	43.70	3.25	2.86	5.86	2.77	2.57
GAW 70-6	ETH	173	208	35	68.50	6.00	9.50	27.40	43.00	3.33	2.88	5.83	2.83	2.67
GAW 125-4	ETH	201	224	23	69.83	6.17	15.30	18.60	56.70	3.43	3.21	5.27	2.37	2.19
GAW 129-2	ETH	184	213	29	84.00	6.83	12.90	51.60	52.70	3.67	2.74	4.98	2.03	1.94
GAW 130-1	ETH	194	219	25	81.13	7.67	13.37	37.60	52.40	3.47	2.57	4.12	2.38	2.22
GAW 130-2	ETH	178	208	30	91.67	6.87	10.43	42.20	53.00	3.43	2.54	4.51	2.41	2.26
GAW 151-8	ETH	191	218	27	73.70	6.47	11.70	33.80	49.40	3.66	2.92	4.53	2.66	2.48
IAR/B/65-1	ETH	182	217	35	57.33	6.17	15.83	16.20	60.00	3.18	2.53	3.83	1.77	1.86
IAR/B/20-3	ETH	194	228	34	67.47	6.80	14.37	11.60	61.70	4.15	3.05	4.36	2.25	2.16
IAR/B/22	ETH	179	212	33	65.67	5.00	7.47	27.00	45.40	3.44	2.79	5.19	2.87	2.50
IAR/B/160-1	ETH	194	222	28	84.83	8.20	15.87	18.20	54.40	3.35	3.57	5.41	2.63	2.50
IAR/B/161-1	ETH	201	224	23	70.80	5.83	16.20	15.40	51.40	3.36	2.83	4.29	2.25	2.28
IAR/B/205-4	ETH	210	230	20	50.00	4.97	13.43	28.00	55.40	3.97	2.70	4.63	2.34	2.20
IAR/B/411-3	ETH	184	215	31	63.43	5.60	13.73	44.60	46.70	3.28	2.45	4.37	2.15	1.94
IAR/B/162-1	ETH	191	223	32	74.43	6.70	13.23	35.80	55.00	3.60	3.00	5.25	2.40	2.31
IAR/B/195-2	ETH	191	223	32	90.83	7.87	13.27	43.00	55.00	3.54	2.81	4.32	1.99	2.25
IAR/B/205-2	ETH	173	206	33	83.33	7.67	11.17	31.40	41.40	3.13	2.68	5.06	2.49	2.62
N41	PAK	172	206	34	62.33	7.00	11.00	33.80	37.40	3.92	3.00	5.92	3.01	3.25
Gopal	IND	173	208	35	83.67	7.93	11.83	23.80	61.70	4.35	4.06	6.90	3.12	3.82

C125	IND	190	217	27	58.00	5.97	12.33	39.60	59.00	3.08	2.53	4.24	1.95	2.09
N125	IND	184	216	32	48.33	4.43	12.93	40.40	51.70	4.82	3.09	5.40	2.66	2.89
C145	IND	183	215	32	87.67	8.83	12.30	44.60	62.00	3.52	2.55	4.66	2.04	2.04
UNA 8341	PER	180	214	34	72.20	6.13	10.60	51.00	51.00	6.19	3.05	4.94	2.55	2.81
UNA 8387	PER	179	214	35	71.33	6.07	10.40	44.20	52.70	5.92	2.96	4.93	2.41	2.64
UNA 8396	PER	181	216	35	70.47	4.47	9.20	40.20	52.40	5.31	2.58	4.54	2.11	2.15
Qingki	CHN	180	220	40	76.63	4.73	12.13	39.20	59.70	5.02	2.41	4.15	1.63	1.80
PI 513174	PAK	175	210	35	61.93	5.70	15.20	22.00	56.70	3.44	1.99	4.22	2.17	2.33
PI 513177	PAK	168	209	41	64.00	6.47	13.50	13.00	48.00	4.92	3.25	6.48	2.93	3.52
PI 513225	PAK	176	209	33	69.03	4.87	8.13	50.40	42.00	5.09	2.63	5.65	3.25	3.27
PI 513234	PAK	173	204	31	60.33	5.33	7.67	35.40	36.00	4.33	2.88	5.89	3.34	3.62
PI 57100	GEO	183	214	31	88.37	5.80	8.40	36.60	51.40	4.21	2.41	4.87	2.58	2.71
PRC92-27	CHN	174	208	34	79.50	8.00	1.12	48.00	35.70	4.29	2.60	5.77	3.14	3.25
WIR 19490	ETH	181	214	33	86.33	9.70	16.57	28.00	51.40	3.57	2.53	5.63	2.86	2.82
WIR 26598	ETH	180	208	28	73.67	8.33	14.33	19.00	53.00	4.97	2.55	5.07	2.65	2.80
SN-26	GEO	180	212	32	82.50	10.63	13.30	25.60	51.00	3.66	2.23	4.76	2.35	2.52
SN-1065	GEO	173	205	32	84.83	7.50	9.17	65.40	43.00	2.94	2.47	5.40	2.70	2.80
SN-2131	GEO	176	209	33	87.67	9.83	12.83	25.60	45.00	3.73	2.74	5.67	2.88	3.08
SN-2158	GEO	175	212	37	92.33	11.67	13.43	26.80	49.70	3.85	2.63	5.63	2.65	2.80
RNB-9	NPL	175	206	31	127.33	8.60	0.53	63.00	36.40	4.26	2.41	6.03	2.64	2.84
RNB-10	NPL	174	206	32	119.93	7.33	0.73	62.20	35.40	3.97	2.51	6.31	2.72	2.86
RNB-119	NPL	173	206	33	90.00	6.50	14.17	56.20	41.40	4.19	2.06	5.28	2.28	2.45
RNB-132	NPL	169	206	37	75.83	5.40	0.00	48.00	43.40	4.56	2.07	5.30	2.12	2.30
RNB-141	NPL	168	206	38	73.50	5.50	9.83	39.60	38.70	4.29	2.11	5.27	2.17	2.44
RNB-169	NPL	170	204	34	57.83	4.17	8.17	32.20	34.40	4.67	2.11	5.43	2.37	2.51
RNB-205	NPL	173	204	31	68.73	4.63	3.20	26.60	33.70	5.00	2.12	5.38	2.03	2.12

RNB-309	NPL	179	208	29	91.67	6.50	4.13	41.20	36.00	4.35	2.30	5.44	2.61	2.64
Nushera	PAK	173	208	35	80.00	4.87	8.60	31.60	43.70	3.80	2.01	4.43	1.83	2.09
2825-1	PAK	180	211	31	68.67	6.93	10.30	55.20	39.40	4.57	2.16	4.89	2.24	2.24
PI 65208	CHN	171	208	37	87.60	5.83	10.97	48.00	36.70	3.41	2.48	5.73	2.51	2.50
PI 69614	CHN	179	212	33	91.00	7.40	9.50	58.80	40.00	3.88	2.73	6.99	2.75	2.81
Georgia	GEO	184	216	32	56.00	6.83	15.37	22.20	57.70	4.71	3.13	4.44	2.11	2.08
SD 5067	KOR	173	208	35	61.33	3.60	7.67	58.20	35.00	4.72	4.30	6.44	3.33	3.10
GEO-MKH-2018-168	GEO	185	216	30	64.50	7.07	11.77	37.20	48.40	3.35	3.50	5.01	2.57	2.47
K230774-2	CHE	179	210	31	76.67	6.43	8.33	39.60	35.00	4.02	3.84	5.85	3.04	2.91
K235060-1	ETH	170	208	38	51.20	4.43	7.27	31.20	37.40	3.94	4.24	5.75	3.01	3.07
K242071-1	PER	179	212	33	64.67	4.83	7.37	41.00	43.40	4.39	3.24	5.19	2.46	2.37
K242071-2	PER	189	216	27	70.83	6.37	12.50	38.40	38.70	3.98	4.19	6.47	3.24	3.11
K242142-2	PAK	179	211	32	91.83	7.53	6.77	49.40	39.70	4.46	4.48	5.98	3.12	3.09
K242143-1	PAK	178	214	36	70.83	7.03	12.40	33.00	45.40	5.23	6.20	4.83	2.23	2.30
K222037-2	TUR	185	209	24	61.50	8.40	11.77	38.40	45.70	3.14	6.97	6.03	3.10	2.97
KSL 180880	KOR	175	210	35	82.83	4.50	8.33	56.80	36.00	3.16	6.54	5.63	2.69	2.65
KSL 180888	KOR	175	209	34	61.13	4.60	5.60	47.20	31.00	3.68	6.54	6.10	2.76	2.64
KSL 180964	KOR	175	208	33	65.50	4.33	8.17	47.20	34.00	4.01	3.39	5.99	3.00	2.92
GEO-HDY-2019-159	GEO	190	221	31	56.17	5.73	13.00	27.80	56.00	3.55	2.69	4.73	2.13	2.16
Gyeongbukyeongcheon-2019-5	KOR	173	207	34	70.17	4.00	9.47	52.80	43.70	3.72	3.21	5.83	2.77	2.68
KSL 191198	KOR	174	207	33	71.67	4.50	7.90	51.20	44.00	3.71	3.06	5.80	2.81	2.70
Pimaek-3	KOR	184	215	31	68.43	3.67	10.07	48.40	37.70	3.68	2.86	5.57	2.60	2.44
Milyangjaeraejong 2ho	KOR	173	207	34	73.50	5.33	7.67	40.40	38.00	3.70	2.94	5.73	2.58	2.42
Oljujukha	KOR	179	214	35	47.93	3.77	3.97	32.20	33.70	4.25	3.08	5.66	2.15	2.14
Aeraemo	KOR	178	213	35	46.90	3.23	2.17	33.80	36.40	5.79	2.72	4.85	2.23	2.11
Gyeongnamhamyang-1985-917	KOR	175	212	37	55.67	3.00	3.83	32.00	30.00	3.24	2.69	5.54	2.31	2.09

Gyeongbukchilgok-1985-962	KOR	175	210	35	62.50	3.83	5.00	48.00	32.40	4.21	3.01	5.45	2.34	2.13
Jeonbukiksan-1985-1094	KOR	174	208	34	66.53	4.27	5.40	45.80	34.70	3.46	2.62	5.22	2.22	2.16
Gyeonghihwaseong-1985-2801	KOR	182	213	31	85.83	3.70	5.40	44.40	41.40	4.26	2.84	5.82	2.59	2.43
Gyeongnamtongyeong-1985-3201	KOR	174	209	35	68.67	4.33	4.33	48.40	32.70	4.47	2.37	6.08	2.31	2.51
Jeonbukokgu-1985-4850	KOR	179	210	31	81.50	4.90	5.73	49.40	32.70	4.25	2.47	6.14	2.07	2.13
Gangwonmyeongju-1985-6585	KOR	182	213	31	91.17	6.00	6.03	55.20	38.70	4.88	2.76	6.60	2.69	2.96
Gangwonsamcheok-1985-6800	KOR	188	216	28	81.17	3.80	5.60	36.60	36.40	4.03	2.32	5.37	2.10	2.42
Gangwonsamcheok-1985-6834	KOR	180	208	28	85.67	5.00	10.50	44.80	38.70	4.20	2.37	6.29	2.53	2.70
Gangwonsamcheok-1985-6967	KOR	174	209	35	80.00	4.67	9.33	55.60	33.70	4.19	2.30	6.15	2.29	2.52
Chungbukokcheon-1985-7311	KOR	179	209	30	78.50	3.90	8.17	48.60	33.00	3.91	2.77	7.18	2.52	2.96
Chungbukokcheon-1985-7350	KOR	175	207	32	76.17	4.67	8.50	53.60	38.00	4.21	3.25	7.49	3.01	3.52
Chungnamseosan-1985-7579	KOR	178	209	31	86.33	6.50	4.67	56.80	36.70	3.45	2.42	5.97	2.31	2.70
Chungnamseocheon-1985-7615	KOR	178	211	33	75.50	6.17	11.00	46.00	35.40	3.54	2.91	7.33	2.90	3.26
Chungnamseocheon-1985-7658	KOR	180	212	32	82.50	3.93	5.70	49.60	35.00	3.19	2.65	6.93	2.70	2.99
Chungnamhongseong-1985-7726	KOR	174	208	34	81.33	5.17	9.70	48.80	37.70	3.45	3.03	7.71	2.67	3.09
Chungnamnonsan-1985-7810	KOR	178	210	32	89.00	5.07	9.40	47.40	35.70	3.37	2.56	5.22	2.51	2.60
Chungnamnonsan-1985-7867	KOR	178	209	31	78.57	5.83	9.13	36.20	31.70	3.70	2.45	5.17	2.39	2.43
Chungnamnonsan-1985-7871	KOR	179	209	30	89.60	4.27	10.37	55.20	32.40	3.35	2.73	5.65	2.81	2.84
Chungnamnonsan-1985-8057	KOR	179	208	29	88.67	6.57	12.37	55.20	32.40	3.50	2.84	6.00	2.85	2.95
Chungnambuyeo-1985-8152	KOR	179	208	29	86.67	4.30	9.93	62.00	32.70	4.36	2.93	6.33	3.36	3.30
CI9980	ETH	183	213	30	75.17	7.93	14.43	23.60	43.00	3.84	2.71	6.07	2.80	2.78
Jeonbukgochang-1985-13356	KOR	175	209	34	59.33	4.40	4.57	55.60	36.00	4.07	2.79	6.30	2.68	2.73
Jnju 87-9	KOR	175	208	33	68.57	4.73	4.70	58.60	34.40	3.11	2.67	4.98	2.35	2.42
Sancheong 87-30	KOR	175	208	33	80.20	5.53	9.97	56.80	28.40	3.26	2.96	5.92	2.98	3.07
Jeonbukgimjae-1989-3410	KOR	175	209	34	59.33	4.33	4.00	47.40	34.70	3.16	2.79	5.21	2.44	2.53
Chungbukokcheon-1995-437	KOR	178	210	32	82.83	4.57	10.07	57.80	35.00	3.47	3.23	5.75	2.92	3.01

Gyeonghihwasseong-1995-440	KOR	178	210	32	64.17	3.57	10.40	36.60	35.00	3.39	3.22	5.89	2.86	2.84
Chungbukokcheon-1995-441	KOR	179	212	33	70.00	4.33	8.83	52.00	36.40	3.67	3.19	5.93	2.88	3.09
Chungnamseosan-1995-445	KOR	175	206	31	77.00	4.67	9.17	60.40	37.40	3.36	3.30	6.31	2.91	3.11
Gyeonghihwasang-1995-447	KOR	175	208	33	60.50	4.33	9.57	47.00	35.70	2.91	3.68	6.70	3.18	3.31
Gyeonghipyeongtaek-1995-448	KOR	175	210	35	74.17	3.33	8.40	51.60	34.40	3.54	3.47	6.77	3.21	3.46
Gyeonghipyeongtaek-1995-449	KOR	176	205	29	72.00	4.17	11.00	54.00	35.40	3.77	3.45	6.66	2.92	3.04
Chungbukokcheon-1995-450	KOR	178	210	32	74.83	3.77	8.80	44.60	36.70	3.21	3.35	6.55	2.86	3.06
Gyeonghiongjin-1995-451	KOR	179	213	34	68.67	3.90	10.33	58.20	37.00	3.89	3.65	6.88	2.75	2.74
Gyeonghihwaseong-1995-453	KOR	179	212	33	62.00	4.23	9.50	45.80	37.40	3.55	3.74	6.39	3.09	3.03
Gangwonmyeongju-1995-457	KOR	179	212	33	58.50	4.87	9.33	55.60	36.40	3.57	3.73	6.34	2.92	2.85
Geumseongjaerae	KOR	180	220	40	50.33	3.63	4.20	41.00	34.70	3.61	3.69	6.31	2.39	2.31
Daegugoyangjaerae-2	KOR	178	209	31	82.83	6.00	11.67	50.20	34.00	3.30	3.04	5.47	2.26	2.12
Jaeraeyukkag (A)	KOR	178	211	33	86.00	4.33	13.53	58.20	33.40	3.58	3.16	6.14	2.56	2.38
CI 6150	CHN	169	206	37	86.67	5.23	8.33	37.40	28.00	3.51	3.16	5.30	2.44	2.54
Abyssinian 1139	ETH	173	209	36	86.00	6.17	12.00	49.00	38.70	3.52	3.02	5.30	2.10	2.24
CIho 3989-2	MNG	180	211	31	100.83	5.57	10.20	39.40	50.00	3.48	3.28	5.68	2.43	2.56
CIho 4169	AFG	173	207	34	77.47	8.53	12.00	20.80	50.40	4.16	2.72	4.30	1.56	1.76
Wase Shu	KOR	175	207	32	87.33	4.10	10.00	46.20	33.00	3.67	3.29	5.65	2.47	2.47
Jou Shirin abi	AFG	185	216	30	65.23	3.63	13.03	39.60	57.70	4.03	3.25	4.98	1.79	1.64
Si Leng Bai Da Mai	CHN	175	208	33	74.13	3.50	9.43	43.40	39.00	3.24	3.37	5.13	2.36	2.32
PI 270611	PER	175	207	32	83.93	5.97	0.00	43.00	42.40	5.04	2.79	4.19	1.82	1.74
PI 270633	PER	182	213	31	86.43	6.23	11.83	30.20	56.00	3.45	3.62	5.34	2.42	2.35
PI 270637	PER	180	212	32	89.77	6.07	11.97	39.60	54.70	3.69	3.78	5.26	2.48	2.48
PI 270675	PER	180	211	31	81.37	6.40	10.87	33.80	51.40	3.39	3.55	5.48	2.43	2.52
PI 270721	PER	182	212	30	86.00	5.73	10.43	39.00	53.40	3.30	2.78	4.67	2.38	2.54
PI 270758	PER	191	221	30	89.47	7.30	12.60	45.00	57.40	3.27	2.67	4.05	1.97	2.03

PI 328905	AFG	194	218	24	69.83	7.80	15.83	23.20	52.00	3.22	2.94	4.56	2.07	2.13
CI 9878	AFG	175	208	33	66.33	5.33	8.00	26.80	39.40	4.21	2.51	4.09	1.97	2.11
CIHo13250	ETH	182	213	31	65.87	8.33	13.43	22.00	46.40	5.15	3.00	4.80	2.03	2.16
Tohoku Shiro Hadaka	CHN	169	208	39	79.83	4.07	3.93	48.80	36.00	3.18	2.65	4.67	2.10	2.04
Shargundik 2	IND	173	206	33	75.00	6.07	0.00	40.80	35.40	4.41	2.47	4.40	1.99	1.83
Jinan Dohadaka	KOR	179	211	32	82.90	3.40	11.10	50.00	34.70	3.58	2.80	4.50	2.01	1.92
Buan Waessalbori	KOR	176	210	34	82.00	4.87	5.40	59.80	32.40	4.46	3.02	4.80	2.16	2.15
CI 4136	AFG	176	204	28	49.17	5.00	7.73	32.00	40.70	4.79	2.70	4.43	2.13	1.91
CIho 3970-1	MNG	185	218	33	73.67	4.67	10.67	33.60	44.70	4.16	2.35	3.85	1.57	1.31
PI 176033	IND	173	205	32	70.33	4.07	7.73	33.80	32.70	3.82	3.26	5.76	2.66	2.56
Pangu	IND	174	207	33	81.33	6.57	9.30	44.00	33.00	4.36	2.73	4.99	2.79	3.07
PI 176122	IND	169	209	40	78.23	5.67	12.33	38.80	49.00	4.16	2.33	4.26	2.24	2.41
PI 202901	CHN	178	208	30	71.07	5.00	8.93	29.80	46.70	4.48	2.98	5.17	2.58	3.05
PI 270619	PER	191	216	25	49.40	3.83	11.67	41.40	46.70	4.18	2.46	4.07	2.18	2.47
PI 270647	PER	182	211	29	73.33	5.77	11.00	35.20	46.70	3.44	2.56	4.72	2.47	2.74
PI 270683	PER	182	211	29	75.70	5.93	11.80	27.40	49.70	3.53	2.66	5.13	2.75	2.92
PI 270704	PER	180	210	30	67.67	4.73	9.53	27.20	39.70	3.46	2.53	4.88	2.45	2.61
PI 270749	PER	180	211	31	72.43	6.20	9.70	33.00	52.00	3.65	2.83	5.24	2.86	3.05
PI 270754	PER	179	209	30	76.67	5.00	9.57	31.60	41.00	3.66	2.53	4.89	2.48	2.67
PI 270729	PER	180	212	32	75.17	6.17	9.47	36.20	39.00	4.29	2.44	4.65	2.34	2.43
1972A	CHE	180	210	30	87.33	6.67	10.37	46.20	36.00	3.12	2.91	5.59	2.89	3.02
ST-96	CHN	166	198	32	71.50	4.73	7.33	24.60	38.00	3.19	2.76	5.61	2.80	2.93
UNA 8461	PER	180	211	31	83.03	4.87	7.27	35.20	42.00	4.32	2.56	3.74	2.25	2.30
PI 447319	CHN	179	216	37	83.80	5.23	10.70	38.40	44.70	4.59	2.64	3.57	2.05	2.03
SN-6	GEO	188	216	28	80.70	8.57	12.17	24.20	62.70	3.30	3.28	4.58	2.87	2.88
GEO-PHJ-2015-3-31	GEO	180	211	31	100.23	6.13	11.47	38.20	49.40	3.27	2.88	4.67	2.88	2.83

PI 61572	GEO	179	209	30	86.83	6.73	11.30	42.80	46.00	3.21	3.05	4.74	2.86	3.02
PI 61508	GEO	175	209	34	71.77	9.23	13.33	19.60	40.70	3.73	3.31	5.48	3.16	3.08
PI 429990	IND	167	201	34	83.33	5.33	2.53	37.80	29.40	3.04	3.14	5.18	3.08	2.91
PI 429610	NPL	178	208	30	91.07	4.40	11.70	56.20	36.70	4.05	3.41	5.00	2.95	2.84
PI 427243	NPL	174	208	34	94.10	5.83	5.50	45.00	49.40	4.44	2.51	3.78	2.23	2.01
Cebada Blanca	PER	179	211	32	94.87	6.97	10.90	53.00	51.00	3.35	3.03	4.71	2.80	2.57
UNA 8338	PER	179	212	33	81.50	6.10	10.23	48.20	52.00	5.84	3.12	4.71	2.66	2.48
PI 270715	PER	179	214	35	101.43	6.80	11.60	46.20	51.40	5.38	2.70	3.83	2.04	1.83
CIho 4181	AFG	175	208	33	64.60	6.37	9.43	50.20	41.70	3.91	2.22	4.35	2.64	2.91
PI 370999	CHE	180	209	29	78.03	8.20	11.70	23.00	39.00	3.28	2.42	4.69	2.61	2.85
PI 371148	CHE	180	211	31	74.37	8.63	11.67	28.00	44.40	3.69	2.58	4.47	2.58	2.77
PI 573706	GEO	179	211	32	100.33	12.00	14.60	31.00	52.70	4.10	2.40	3.83	2.13	2.37
PI 574091	NPL	176	206	30	85.50	4.97	11.07	48.00	34.70	4.64	2.72	4.27	2.37	2.53
PI 477805	PER	178	214	36	93.00	5.80	10.83	37.80	55.40	5.84	1.88	3.07	1.66	1.74
PI 477851	PER	175	210	35	91.10	5.07	10.03	56.40	45.70	4.96	2.07	3.88	2.18	1.92
PI 510561	PER	181	216	35	71.50	3.60	11.00	34.20	44.70	4.47	2.98	4.39	2.22	2.20
PI 342163	TUR	185	209	24	72.60	9.70	10.47	26.20	51.00	3.41	2.19	4.11	2.08	2.01
CIho 6962	AFG	180	208	28	58.23	5.27	10.53	42.60	47.40	3.90	2.50	4.30	2.31	2.16
PI 270707	PER	180	212	32	78.33	6.33	8.33	57.60	37.70	4.84	2.51	4.85	2.53	2.33
PI 296470	ETH	178	209	31	67.17	10.07	15.00	21.40	51.00	4.03	2.16	4.05	2.04	1.87
PI 328189	TUR	189	216	27	83.43	6.23	11.53	33.80	47.70	3.46	2.77	5.37	3.00	3.50
PI 510558	PER	178	210	32	96.00	7.30	0.00	50.00	40.70	4.13	2.58	5.60	3.14	3.32
PI 559514	NPL	166	200	34	99.27	6.10	7.90	51.40	35.70	3.91	3.09	6.04	3.62	3.80
OUI 426	IND	166	199	33	91.67	5.30	6.90	44.40	33.00	3.26	2.49	4.90	3.02	3.29
PI 270618	PER	175	208	33	90.07	5.07	10.87	37.60	43.40	4.21	2.39	4.74	2.74	2.91
PI 27829	GEO	182	207	25	78.00	6.93	9.97	51.20	38.70	3.53	2.80	5.60	3.37	3.59

CIho 9940	CHN	174	207	33	87.33	5.63	10.27	48.40	40.40	4.19	2.74	5.91	3.31	3.04
PI 477832	PER	175	213	38	82.07	6.47	10.67	45.20	53.00	5.92	3.06	5.84	3.16	3.22
PI 366428	AFG	170	202	32	83.07	8.43	11.67	20.60	46.40	3.58	2.58	4.96	2.53	2.42
PI 69607	CHN	174	206	32	111.40	7.67	9.13	50.60	38.00	2.95	3.13	6.22	3.31	3.14
OUI 420	IND	168	204	36	82.00	7.00	8.43	43.20	43.70	3.93	2.60	5.48	2.84	2.62
PI 370970	CHE	178	210	32	85.50	7.13	11.70	52.80	32.40	3.75	3.23	7.17	3.83	3.71
Betaone	KOR	180	215	35	72.00	4.57	13.67	49.20	35.40	8.41	4.00	8.88	5.67	5.55
Saechalssalbori	KOR	180	208	28	39.50	3.10	8.67	25.60	30.40	4.29	3.68	7.20	3.97	4.16
Heuknuri	KOR	176	208	32	71.17	5.90	10.13	48.80	39.00	4.06	3.08	5.71	2.98	3.22
Heukdahyang	KOR	173	206	33	66.17	5.10	11.00	43.60	45.00	3.76	3.16	6.60	3.58	3.71
Saessalbori	KOR	174	208	34	47.67	4.23	5.43	52.00	34.00	3.75	2.91	5.79	3.02	3.12
LSD$_{(0.05)}$		-	-	-	4.60	1.20	1.79	7.34	1.52	0.16	0.16	0.29	0.14	0.16

ABTS: ABTS$^{\bullet+}$ scavenging activity (in mg AAE/100g), AL: Awn length (in mm), CL: Culm length (in cm), DH: Days to heading (in days), DM: days to maturity (in days), DHM: Days from heading to maturity (in days), DPPH: DPPH$^{\bullet}$ scavenging activity (in mg AAE/g), GPP: Number of grains per panicle (*n*), RP: Reducing power (in mg AAE/g), SL: Spike length (in cm), TGW: One-thousand grains weight (in g), TPC: Total phenolic content (in mg GAE/g), β-GL: β-glucan content (in g/100g). Coutry codes representations are similar to those shown in Table S1 foot notes.

Table S4. Average values of top performing accessions relative to the control cultivars.

Early maturing accessions		
Name	Origin	DM
ST-96	CHN	198
OUI 426	IND	199
PI 559514	NPL	200
PI 429990	IND	201
PI 366428	AFG	202
OUI 420	IND	204
PI 328499	GRC	204
RNB-205	NPL	204
PI 513234	PAK	204
RNB-169	NPL	204
CI 4136	AFG	204
SN-1065	GEO	205
PI 316806	ETH	205
Gyeongipyeongtaek-1995-449	KOR	205
PI 176033	IND	205
GAW 89-11	ETH	205
GAW 90-5	ETH	205
MOR 8/4	MAR	205
**Accessions with longer AL**		
Name	Origin	AL
HVS 355	LBN	21.30
CI 6222	TUR	21.30
HVS 366	LBN	20.37
PI 466252	LBN	20.13
**Accessions with higher GPP**		
Name	Origin	GPP
E 272/2	ETH	67.40
SN-1065	GEO	65.40
E 282/2	ETH	64.20
RNB-9	NPL	63.00
RNB-10	NPL	62.20
**Gochang**-87-29	KOR	62.00
**Chungnambuyo**-1985-8152	KOR	62.00

Accessions with both longer CL and SL			
Name	Origin	CL	SL
GRA2621	GRC	115.17	13.03
GRA1015	UKR	109.07	13.63
GRA2256	TJK	108.67	14.40
PI 573706	GEO	100.33	12.00
**Accessions with higher TPC**			
Name	Origin	TPC	
K222037-2	TUR	6.97	
KSL 180880	KOR	6.54	
KSL 180888	KOR	6.54	
GRA2621	GRC	6.22	
K242143-1	PAK	6.20	
GRA2256	TJK	4.85	
PI 304357	PRT	4.51	
K242142-2	PAK	4.48	
UZB-BHJ-2002-23-2	UZB	4.39	
PI 204875	TUR	4.37	
SD 5067	KOR	4.30	
Soncheon 87-16	KOR	4.30	
K235060-1	ETH	4.24	
K242071-2	PER	4.19	
UZB-BHJ-2002-12-3	UZB	4.16	
GRA1015	UKR	4.14	
PI 204705	TUR	4.10	
Castelar-034	ARG	4.09	
Castelar-668	ARG	4.07	
Gopal	IND	4.06	
**Accessions with higher β-GL**			
Name	Origin	β-GL	
PI 235639	DEU	7.67	
IG 38956	ISR	7.08	
**Accessions with higher ABTS**			
Name	Origin	ABTS	
GRA2621	GRC	13.54	

**Gwangsan** 87-11	KOR	61.80
WIR398	MNG	61.60
**chungnamseosan**-1995-445	KOR	60.40

GRA2256	TJK	10.65
UZB-BHJ-2002-23-2	UZB	9.93
PI 204875	TUR	9.83
GRA1015	UKR	8.92

Accessions with higher TGW

Name	Origin	TGW (g)
GAW 72-11	ETH	68.00
GAW 26-2	ETH	66.00
Shirok Kapo	IND	65.00
PI 282649	ISR	63.70
CI 6221	TUR	63.40
PI 328488	GRC	62.70
SN-6	GEO	62.70
GAW 64-4	ETH	62.70
PI 328521	GRC	62.40
C145	IND	62.00
MOR 13/4	MAR	62.00
H-2185	ETH	62.00

Accessions with higher DPPH and RP

Name	Origin	DPPH	RP
GRA2621	GRC	6.24	7.86
GRA2256	TJK	4.20	6.13
PI 204875	TUR	3.85	4.05
PI 370970	CHE	3.83	3.71
UZB-BHJ-2002-23-2	UZB	3.81	3.82
PI 328420	GRC	3.70	
PI 304357	PRT	3.69	
PI 559514	NPL	3.62	
Suncheon-87-16	KOR	3.58	

Performance of control cultivars

**Name**	**DM**	**CL**	**SL**	**AL**	**GPP**	**TGW**	TPC	b-GL	ABTS	DPPH	RP
Betaone	215	72.0	4.6	13.7	49.2	35.40	4.00	8.41	8.88	5.67	5.55
Saechalssalbori	208	39.5	3.1	8.7	25.6	30.40	3.68	4.29	7.20	3.97	4.16
Heknuri	208	71.2	5.9	10.1	48.8	39.00	3.08	4.06	5.71	2.98	3.22
Heukdahyang	206	66.2	5.1	11.0	43.6	45.00	3.16	3.76	6.60	3.58	3.71
Saessalbori	208	47.7	4.2	5.4	52.0	34.00	2.91	3.75	5.79	3.02	3.12

ABTS: $ABTS^{\bullet+}$ scavenging activity (in mg AAE/100g), AL: Awn length (in mm), CL: Culm length (in cm), DH: Days to heading (in days), DM: days to maturity (in days), DHM: Days from heading to maturity (in days), DPPH: $DPPH^{\bullet}$ scavenging activity (in mg AAE/g), GPP: Number of grains per panicle (*n*), RP: Reducing power (in mg AAE/g), SL: Spike length (in cm), TGW: One-thousand grains weight (in g), TPC: Total phenolic content (in mg GAE/g), β-GL: β-glucan content (in g/100g). Coutry codes representations are similar to those shown in Table S1 foot notes.

Table S5. Effect of origin on qunatittaive agronomical traits and biochemical parameters in global barley accessions grown in Korea.

		Agronomical traits								Biochemical traits				
**Origin**	**Values**	**DH (Days)**	**DM (Days)**	**DHM (Days)**	**CL (cm)**	**SL (cm)**	**AL (mm)**	**GPP (n)**	**TGW (g)**	***β*-GL**	**TPC**	**DPPH**	**ABTS**	**RP**
AFG	Min	170.00	202.00	24.00	34.90	3.63	4.33	19.20	4.00	0.46	2.22	1.56	4.09	1.64
	Max	194.00	224.00	34.00	83.07	8.83	15.83	56.00	57.70	4.79	3.49	2.94	7.43	2.91
	Mean	180.27 ^{bcd}	210.82 ^{cde}	30.46 ^{efg}	62.36 ^{f}	6.59 ^{bc}	9.64 ^{def}	32.06 ^{efg}	39.92 ^{efg}	3.26 ^{fgh}	2.82 ^{bcd}	2.24 ^{e}	4.95 ^{bc}	2.27 ^{f}
	SD	7.16	6.22	2.97	15.06	1.61	3.34	12.47	16.22	1.37	0.37	0.38	0.94	0.39
CHE	Min	175.00	206.00	22.00	53.30	4.17	0.00	12.80	32.40	2.85	2.09	2.09	4.09	1.81
	Max	201.00	224.00	36.00	95.67	11.23	17.77	52.80	57.70	4.26	3.85	3.83	7.17	3.71
	Mean	184.45 ^{ab}	213.65 ^{bcd}	29.18 ^{g}	79.07 ^{bcd}	8.35 ^{a}	13.07 ^{a}	26.36 ^{g}	46.61 ^{bcd}	3.50 ^{efg}	2.83 ^{bcd}	2.78 ^{bc}	5.13 ^{bc}	2.75 ^{b}
	SD	5.81	3.76	3.42	9.16	1.41	2.62	8.59	4.90	0.34	0.45	0.29	0.61	0.28
CHN	Min	166.00	198.00	30.00	62.83	3.50	1.12	20.00	28.00	2.95	2.41	1.63	3.57	1.80
	Max	187.00	222.00	40.00	111.40	8.00	13.67	58.80	59.70	5.39	3.37	3.31	6.99	3.25
	Mean	175.29 ^{ef}	210.00 ^{def}	34.71 ^{ab}	80.89 ^{ab}	5.72 ^{c}	8.78 ^{efg}	41.29 ^{bc}	42.36 ^{def}	3.94 ^{bcde}	2.80 ^{cd}	2.51 ^{cde}	5.22 ^{bc}	2.52 ^{bcdef}
	SD	5.32	5.96	2.79	11.58	1.32	3.09	10.31	8.63	0.72	0.26	0.49	0.88	0.50
ETH	Min	169.00	205.00	20.00	50.00	3.77	7.27	11.60	37.40	2.81	1.79	1.72	3.27	1.63
	Max	210.00	230.00	45.00	107.13	10.23	19.37	67.40	68.00	5.26	4.24	3.05	6.27	3.07
	Mean	183.32 ^{bc}	214.28 ^{bc}	30.94 ^{defg}	74.02 ^{bcde}	7.00 ^{b}	13.41 ^{a}	30.67 ^{efg}	51.07 ^{ab}	3.83 ^{cde}	2.77 ^{cd}	2.42 ^{de}	4.85 ^{c}	2.32 ^{ef}
	SD	8.88	6.86	4.05	11.15	1.56	2.97	12.36	6.76	0.57	0.43	0.33	0.64	0.30
GEO	Min	173.00	205.00	25.00	56.00	5.73	8.40	19.60	38.70	2.94	2.23	2.11	3.83	2.08
	Max	190.00	221.00	37.00	100.33	12.00	15.37	65.40	62.70	4.71	3.50	3.37	5.67	3.59
	Mean	180.64 ^{bcd}	212.00 ^{cde}	31.29 ^{defg}	80.73 ^{ab}	8.19 ^{a}	12.15 ^{abc}	33.87 ^{def}	49.46 ^{bc}	3.65 ^{def}	2.82 ^{bcd}	2.66 ^{bcd}	4.96 ^{bc}	2.74 ^{bc}
	SD	4.85	4.11	2.66	13.70	2.07	1.91	12.18	6.38	0.45	0.37	0.37	0.52	0.39
GRC	Min	169.00	204.00	28.00	56.90	4.17	5.60	16.80	11.00	0.43	2.74	2.12	4.28	2.15
	Max	201.00	229.00	38.00	115.17	13.03	18.23	48.40	62.70	3.92	6.22	6.24	13.54	7.86
	Mean	184.27 ^{b}	215.91 ^{ab}	31.55 ^{cdefg}	79.56 ^{bc}	6.24 ^{bc}	12.65 ^{ab}	37.18 ^{cde}	51.24 ^{ab}	3.04 ^{gh}	3.32 ^{a}	3.22 ^{a}	6.34 ^{a}	3.21 ^{a}
	SD	8.77	6.53	3.09	14.75	2.35	3.15	9.28	13.56	0.91	0.95	1.05	2.45	1.50
IND	Min	166.00	199.00	27.00	48.33	4.07	0.00	23.80	29.40	3.04	2.33	1.93	4.05	1.72
	Max	199.00	229.00	42.00	91.67	8.83	13.87	50.40	65.00	4.82	4.06	3.12	6.90	3.82
	Mean	176.64 ^{def}	210.57 ^{de}	33.93 ^{abc}	73.63 ^{bcde}	6.43 ^{bc}	9.57 ^{def}	39.51 ^{cd}	47.38 ^{bcd}	4.05 ^{abcd}	2.76 ^{cd}	2.52 ^{bcde}	4.98 ^{bc}	2.57 ^{bcdef}
	SD	9.17	8.03	3.67	12.93	1.37	3.97	6.59	12.40	0.59	0.46	0.45	0.74	0.59
KOR	Min	173.00	205.00	26.00	46.90	3.00	2.17	24.40	28.40	2.91	2.30	2.01	4.50	1.92
	Max	188.00	220.00	40.00	91.17	9.30	14.83	62.00	51.70	5.79	6.54	3.58	7.71	3.52
	Mean	177.27 ^{def}	209.86 ^{ef}	32.59 ^{bcde}	74.16 ^{bcde}	4.73 ^{d}	8.07 ^{fg}	49.26 ^{a}	35.88 ^{g}	3.71 ^{def}	3.19 ^{abc}	2.68 ^{bcd}	6.03 ^{a}	2.69 ^{bcd}
	SD	3.12	2.63	2.32	11.02	1.21	2.98	8.54	3.83	0.50	0.75	0.34	0.65	0.38

MAR	Min	171.00	205.00	27.00	46.17	4.83	9.50	16.20	45.00	2.87	2.52	2.13	4.17	1.98
	Max	189.00	216.00	36.00	82.57	7.30	17.10	47.20	62.00	4.18	3.51	2.99	5.83	2.86
	Mean	178.82 ^{cde}	211.55 ^{cde}	32.73 ^{abcde}	62.02 ^{f}	5.99 ^{c}	13.48 ^{a}	30.20 ^{efg}	56.35 ^{a}	3.69 ^{def}	2.96 ^{abc}	2.43 ^{de}	4.89 ^{bc}	2.39 ^{def}
	SD	5.47	3.53	2.56	9.31	0.82	2.18	9.63	6.09	0.38	0.35	0.25	0.45	0.27
MNG	Min	170.00	207.00	16.00	46.10	3.50	0.00	18.60	36.70	3.40	2.24	1.57	3.82	1.31
	Max	212.00	228.00	41.00	100.83	8.13	17.20	61.60	60.00	4.71	3.28	3.10	5.68	3.16
	Mean	184.58 ^{ab}	215.92 ^{ab}	31.33 ^{defg}	70.50 ^{def}	5.75 ^{c}	10.38 ^{bcde}	41.27 ^{bc}	45.90 ^{bcd}	4.06 ^{abcd}	2.77 ^{cd}	2.47 ^{de}	4.89 ^{bc}	2.41 ^{cdef}
	SD	10.27	6.12	5.66	14.12	1.34	3.95	11.97	6.30	0.42	0.37	0.41	0.67	0.47
NPL	Min	166.00	200.00	29.00	57.83	4.17	0.00	26.60	33.70	3.32	2.06	2.03	3.78	2.01
	Max	179.00	214.00	38.00	127.33	8.60	14.17	63.00	49.40	5.00	3.41	3.62	6.31	3.80
	Mean	173.36 ^{f}	206.57 ^{f}	33.21 ^{abcd}	88.25 ^{a}	5.90 ^{c}	6.81 ^{g}	46.64 ^{ab}	38.04 ^{fg}	4.32 ^{ab}	2.52 ^{d}	2.60 ^{bcd}	5.38 ^{b}	2.65 ^{bcde}
	SD	3.87	3.06	2.76	18.09	1.19	4.39	10.33	4.12	0.42	0.42	0.46	0.68	0.42
PAK	Min	168.00	204.00	31.00	60.33	4.50	6.27	13.00	36.00	3.08	1.99	1.83	4.22	2.09
	Max	180.00	222.00	42.00	91.83	7.67	15.20	55.20	56.70	5.23	6.20	3.34	6.48	3.62
	Mean	175.27 ^{ef}	210.36 ^{de}	35.09 ^{a}	72.08 ^{cde}	6.17 ^{bc}	10.23 ^{cdef}	36.98 ^{cde}	43.79 ^{cde}	4.20 ^{abc}	3.05 ^{abc}	2.69 ^{bcd}	5.32 ^{bc}	2.79 ^{b}
	SD	3.60	4.46	3.48	9.87	1.10	2.83	12.15	5.61	0.70	1.20	0.48	0.69	0.54
PER	Min	175.00	207.00	25.00	49.40	3.60	0.00	27.20	37.70	3.27	1.88	1.66	3.07	1.74
	Max	191.00	221.00	38.00	101.43	7.30	12.60	57.60	57.40	6.19	4.19	3.24	6.47	3.32
	Mean	180.73 ^{bcd}	212.57 ^{bcde}	31.83 ^{cdef}	79.70 ^{abc}	5.83 ^{c}	9.41 ^{def}	41.05 ^{bc}	47.62 ^{bcd}	4.45 ^{a}	2.83 ^{bcd}	2.47 ^{de}	4.78 ^{c}	2.50 ^{bcdef}
	SD	4.32	2.93	2.83	10.90	0.94	3.38	8.11	5.82	0.96	0.48	0.37	0.68	0.41
TUR	Min	177.00	209.00	24.00	23.50	3.70	2.33	18.80	3.00	0.30	2.19	2.08	4.11	2.01
	Max	213.00	238.00	39.00	95.40	13.17	21.30	47.60	63.40	4.31	6.97	3.85	9.83	4.05
	Mean	189.07 ^{a}	219.00 ^{a}	29.93 ^{fg}	67.39 ^{ef}	8.22 ^{a}	11.31 ^{abcd}	28.37 ^{fg}	43.91 ^{cde}	2.99 ^{h}	3.25 ^{ab}	2.81 ^{b}	6.10 ^{a}	2.64 ^{bcde}
	SD	9.51	9.24	4.11	17.10	2.66	5.10	8.06	19.65	1.40	1.20	0.42	1.53	0.53

ABTS: $ABTS^{\bullet+}$ scavenging activity (in mg AAE/100g), AL: Awn length (in mm), CL: Culm length (in cm), DH: Days to heading (in days), DM: days to maturity (in days), DHM: Days from heading to maturity (in days), DPPH: $DPPH^{\bullet}$ scavenging activity (in mg AAE/g), GPP: Number of grains per panicle (*n*), RP: Reducing power (in mg AAE/g), SL: Spike length (in cm), TGW: One-thousand grains weight (in g), TPC: Total phenolic content (in mg GAE/g), β-GL: β-glucan content (in g/100g). Coutry codes representations are similar to those shown in Table S1 foot notes.
